# Measuring strengths and weaknesses in dimensional psychiatry

**DOI:** 10.1111/jcpp.13104

**Published:** 2019-08-18

**Authors:** Lindsay M. Alexander, Giovanni A. Salum, James M. Swanson, Michael P. Milham

**Affiliations:** ^1^ Center for the Developing Brain Child Mind Institute New York NY USA; ^2^ Department of Psychiatry Universidade Federal do Rio Grande do Sul Porto Alegre Brazil; ^3^ Department of Pediatrics University of California, Irvine Irvine CA USA; ^4^ Center for Biomedical Imaging and Neuromodulation Nathan S. Kline Institute for Psychiatric Research Orangeburg NY USA

**Keywords:** Questionnaires, rating scales, methodology

## Abstract

**Background:**

The Extended Strengths and Weaknesses Assessment of Normal Behavior (E‐SWAN) reconceptualizes each diagnostic criterion for selected DSM‐5 disorders as a behavior, which can range from high (strengths) to low (weaknesses). Initial development focused on Panic Disorder, Social Anxiety, Major Depression, and Disruptive Mood Dysregulation Disorder.

**Methods:**

Data were collected from 523 participants (ages 6–17). Parents completed each of the four E‐SWAN scales and traditional unidirectional scales addressing the same disorders. Distributional properties, Item Response Theory Analysis (IRT), and Receiver Operating Characteristic (ROC) curves were used to assess and compare the performance of E‐SWAN and traditional scales.

**Results:**

In contrast to the traditional scales, which exhibited truncated distributions, all four E‐SWAN scales had symmetric distributions. IRT analyses indicate the E‐SWAN subscales provided reliable information about respondents throughout the population distribution; traditional scales only provided reliable information about respondents at the high end of the distribution. Predictive value for DSM‐5 diagnoses was comparable to prior scales.

**Conclusions:**

E‐SWAN bidirectional scales can capture the full spectrum of the population distribution of behavior underlying DSM disorders. The additional information provided can better inform examination of inter‐individual variation in population studies, as well as facilitate the identification of factors related to resiliency in clinical samples.

## Introduction

Myriad questionnaires are available for measuring psychiatric illness dimensionally (Achenbach & Edelbrock, 1983; Goodman, 2001). However, the vast majority are based on detection of the presence of problematic behaviors and symptoms. Although useful from a clinical perspective, the tendency to focus on only ‘one end’ of the distribution (i.e., the pathologic trait range) limits the ability of such tools to distinguish individuals from one another in less symptomatic or nonaffected segments of the population (i.e., the distribution is truncated; Axelrud et al., 2017; Greven, Buitelaar, & Salum, 2018). This failure to consider differences in strengths among individuals is particularly problematic for psychiatric research, where efforts to model brain–behavior relationships are increasingly turning to broader community and transdiagnostic samples (Insel et al., 2010).

Some assessments have attempted to address this problem (McDowell, 2006). The Strengths and Weaknesses Assessment of ADHD Symptoms and Normal Behavior (SWAN) provides a potentially valuable model for bidirectional questionnaire design (Swanson et al., 2012). Rather than attempting to quantify only the presence of ADHD symptoms, the SWAN probes a range of behaviors to identify relative strengths (i.e., abilities, which are indicative of adaptive behavior) and weaknesses (i.e., disabilities, which are indicative of problems requiring clinical attention, such as ADHD). This was accomplished by: (a) converting each DSM‐IV ADHD symptom into a behavior and (b) expanding the typical 4‐point scale of symptom presence (‘not at all’ to ‘very much’) to a 7‐point scale (‘far below average’ to ‘far above average’). Numerous published studies have demonstrated that the SWAN generates bidirectional distributions that are near‐normal (Arnett et al., 2013; Lakes, Swanson, & Riggs, 2012; Young, Levy, Martin, & Hay, 2009)^.^ Importantly, among individuals with ADHD symptomatology (i.e., a clinical sample), there is generally a high degree of agreement between the SWAN and traditional scales (Greven et al., 2018).

Here, we report on the initial design and feasibility testing of the Extended Strengths and Weaknesses Assessment of Normal Behavior (E‐SWAN) – a framework that extends the general methodology of the SWAN to other psychiatric disorders. Consistent with the SWAN, the clinical wisdom embodied in the DSM‐5 was taken as the departure point to develop each E‐SWAN scale. Four disorders were chosen to provide a sampling of challenges that can arise in the conversion of DSM symptoms to dimensional probes. Major Depressive Disorder and Social Anxiety were chosen for their high prevalence in the general population (Grant et al., 2005; Kessler et al., 2003). Disruptive Mood Dysregulation Disorder (DMDD) was chosen as this new disorder in DSM‐5 does not have empirically defined criteria or many valid measures for assessing symptoms (Vidal‐Ribas, Brotman, Valdivieso, Leibenluft, & Stringaris, 2016). Finally, Panic Disorder was chosen to determine the feasibility of applying this framework to a disorder with physiological symptoms (American Psychiatric Association, 2013).

The present work makes use of initial data obtained in the Child Mind Institute Healthy Brain Network (CMI‐HBN) sample (ages: 6–17; *N* = 523) that enabled comparison of E‐SWAN results with those obtained using equivalent unidirectional questionnaires in the same individuals. Item response theory analyses, showing in what area of the latent trait the scale scores are reliable enough to provide information about the subjects, are included to demonstrate the added value of the information obtained via the E‐SWAN. Additionally, we obtained informant and self‐report data via the Prolific Academic platform to verify the bidirectional distributional properties of the E‐SWAN in an independent sample with distinct characteristics (*n* = 250).

## Methods

### Overview

**Figure 1:**
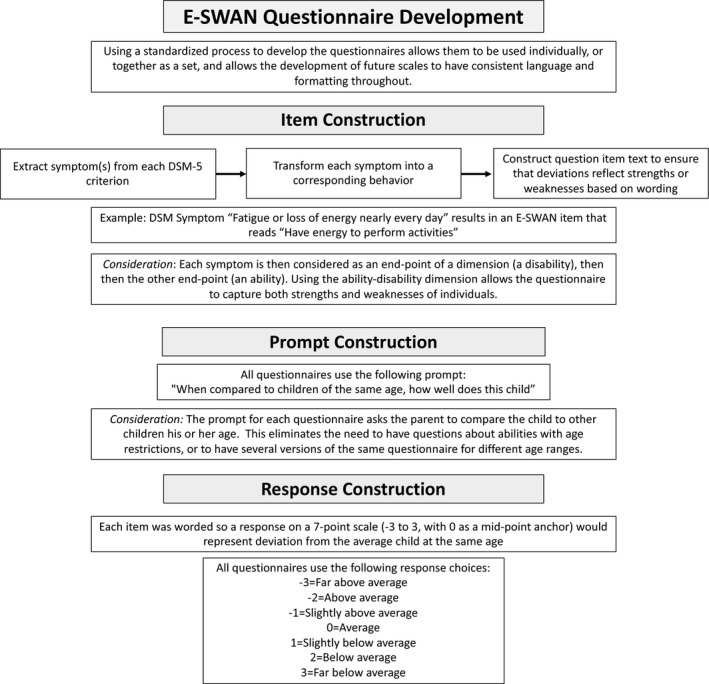
E‐SWAN Questionnaire Development Workflow. Workflow diagram detailing the steps followed for developing the items of each E‐SWAN questionnaire

The present work focused on the development and testing of four E‐SWAN questionnaires using a uniform method based on the method previously employed for construction of the SWAN (see Figure 1). Each questionnaire, corresponded to 1 of 4 following DSM diagnoses: (1) Depression, (2) Social Anxiety, (3) Panic Disorder, (4) DMDD. The following sections describe the questionnaire construction process.

### Questionnaire construction: process

*First*, each DSM‐5 criterion was broken down to reflect core symptoms of the DSM‐5 disorders. *Second*, each specific symptom was transformed into its underlying ability or behavior, that is, the ability/behavior that when impaired or dysfunctional gives rise to the symptom. *Lastly*, each item was worded to be answered on a 7‐point scale representing deviation from children of the same age, following the statement: ‘When compared to children of the same age, how well does this child…’ Results from this process were discussed by a committee of experts and final versions circulated to experienced clinicians for comments (Figure 1). For example, the DSM‐5 Depression symptom ‘Fatigue or loss of energy nearly every day’ results in an E‐SWAN item that reads ‘Have energy to perform activities’, and the DSM‐5 Social Anxiety symptom ‘Marked fear or anxiety about one or more social situations in which the individual is exposed to possible scrutiny by others’ results in an E‐SWAN item that reads ‘Tolerate feelings of anxiety in social situations.’ See Figure S1 in the Supporting Information for additional example questions.

### Questionnaire construction: considerations

#### Level of detail and nuance

When converting DSM criteria to behaviors, we made efforts to ensure that question items capture the level of detail and nuance of the original DSM criteria. This is essential as even slight changes can impact the interpretation of and responses to an item. An example of the importance of this consideration is in the DMDD questionnaire. A key criterion of a DMDD diagnosis is that the behaviors must be present in more than on setting. Initially, we indicated in each question that the behavior being rated must have taken place in more than one setting. However, we found this to be problematic, as it required parents to think about a behavior over several settings at once and was not informative as to the specific setting(s) in which the behavior is actually taking place. As a result, we changed the questionnaire to ask each question separately for each of the settings (home, school, and with friends; Figure S1).

#### Multiple phrases versus single

DSM diagnoses differ in number and complexity of criteria. Some DSM diagnoses have simple, clearly defined criteria, such as a symptom count, while other diagnoses contain long phrases capturing many symptoms or multiple contexts in one criterion. For example, when looking at ADHD, Depression or Social Anxiety, each criterion is generally a single symptom. In contrast, for DMDD, each criterion encapsulates multiple symptoms (e.g., ‘Severe recurrent temper outbursts manifested verbally and/or behaviorally that are grossly out of proportion in intensity or duration to the situation or provocation’). In the first step of the E‐SWAN construction process, complex criteria such as this are broken down into multiple items (Figure S1).

#### Conditional criteria

Some DSM diagnoses have conditional criteria that cannot easily be translated into abilities or strengths. For example, Panic Disorder criteria are mostly physiological symptoms experienced during a panic attack. To address this, we first developed three questions focused on the presence and severity of panic attacks (phrased as ‘moments of intense fear or discomfort’). We then ask the parent to rate how well their child is able to regulate the physiological symptoms while experiencing ‘a moment of intense fear or discomfort’. This allows us to potentially capture what prevents a panic attack in one individual in the same context that elicits a panic attack in another individual.

#### Question development guidelines

To promote standardization across current and future E‐SWAN assessments, we focused on ensuring that all questions met specific criteria. In particular, we focused on three criteria: clarity, precision, and general applicability. Clarity means that each item is straightforward and easy to understand – not vague, confusing, or complex. To meet this goal, we used simple language characteristic of a sixth‐grade reading level. Precision means that each question is specific, asking only about one behavior in one setting. We did not include multiple behaviors in one item. To meet this goal, several of the original questions were broken down into multiple questions. General applicability means that the questions do not require cultural or contextual knowledge. To meet this goal, the scale was constructed including input for item development and review from individuals with different cultural backgrounds.

### Participants

#### Child Mind Institute – Healthy Brain Network (CMI‐HBN)

Data were collected from 523 participants (ages 6.0–17.0 year old, mean age 10.3; M:F 309:214) of the CMI‐HBN (Alexander et al., 2017), which is designed to recruit a sample of 10,000 children and adolescents from the New York City area, collected using a community self‐referred model that recruits participants based on the presence of behavioral concerns. As part of the CMI‐HBN protocol, parents of participants completed all four E‐SWAN scales. Additionally, they completed traditionally designed instruments to assess these same disorders, including the Mood and Feelings Questionnaire (MFQ; Angold, Costello, & Messer, 1995), a measure of depression; the Screen for Child Anxiety and Related Disorders (SCARED; Birmaher et al., 1997), which includes subscales for social anxiety and panic disorder; the Affective Reactivity Index (ARI; Stringaris et al., 2012), a measure of irritability; and the Strengths and Difficulties Questionnaire (SDQ; Goodman, 2001), which includes a subscale for hyperactivity problems. DSM‐5 diagnoses were established using a computerized version of the Kiddie Schedule for Affective Disorders and Schizophrenia (KSADS; Anon, 2017) that was administered by licensed/license‐eligible clinicians. The CMI‐HBN protocol was approved by the Chesapeake Institutional Review Board ( https://www.chesapeakeirb.com/). Prior to collecting data, written informed consent is obtained from participants’ legal guardians and written assent obtained from the participant and all methods were performed in accordance with the relevant guidelines and regulations.

#### Prolific academic sample

To confirm the generality of distributional properties for the E‐SWAN, additional data were collected from 250 parents through Prolific Academic (PA; an online crowdsourced data collection tool; https://www.prolific.ac/). Users were screened based on having a child in the 6.0–17.0 age range. Parents then completed four questionnaires anonymously through a Google survey. This sample had a younger mean age (8.75) than the CMI‐HBN sample, and a more balanced male to female ratio, with slightly more females (115:135). These respondents completed the E‐SWAN questionnaires only and were given a small monetary compensation. Additionally, we collected 250 adult self‐report surveys (ages 18–67 years, mean age 33.64; M:F 139:111). Because all data were collected anonymously, through a platform designed for research data collection, no IRB oversight was required for this sample. Exemption from IRB oversight was approved by the Chesapeake Institutional Review Board. Participants using the Prolific Academic website are required to agree to the Terms of Service notification ( https://prolific.ac/assets/docs/Participant_Terms.pdf) before being allowed to complete surveys. Per the IRB exemption, no additional informed consent was required.

### Statistical analysis

#### E‐SWAN distributional properties

For each of the bidirectional E‐SWAN scales and corresponding traditional unidirectional scales, mean, median, skewness, and kurtosis were calculated. Distributional properties were calculated for both the CMI‐HBN and PA samples.

#### Testing correspondence between E‐SWAN and traditional scales

Using the CMI‐HBN sample, for each of the E‐SWAN scales, and its unidirectional counterpart, we calculated (a) Cronbach's alpha coefficients for individual scale items, and between scores on the corresponding scales, to measure internal consistency within and between scales, and (b) Omega reliability for each of the scales, (c) Pearson correlation coefficients. All questionnaire items for each domain were included in these analyses. Internal consistency analysis was performed without scaling. To further examine the added value of measuring both strengths and weaknesses, for each questionnaire, we subdivided participants into those with mean E‐SWAN scores greater for strengths, or negative scores, based on the scoring convention (E‐SWAN mean score < 0) and those with mean E‐SWAN scores greater for weaknesses, or positive scores (E‐SWAN mean score > 0), and then calculated Cronbach's alpha and Pearson correlation coefficients again for each of the two groups. Finally, we tested whether correlations between E‐SWAN scales and traditional scales vary as a function of E‐SWAN score using quantile regression, which assesses whether the correlation between two instruments is the same for varying levels of a latent trait (Koenker & Bassett, 1978).

#### Psychometric analysis

Next, for the CMI‐HBN sample, we evaluated the performance of individual items from the E‐SWAN and their traditional counterparts in measuring the latent trait using Item Response Theory (IRT) and Confirmatory Factor Analysis (CFA). We followed three analytical approaches.

#### Approach 1

All questions for a given domain together (e.g., E‐SWAN Depression and MFQ together) as a single unidimensional IRT model. This analysis aims to directly compare the level of information provided by unidirectional and bidirectional scales using the same latent trait. Prior to this analysis, ‘sufficient’ unidimensionality of the total item pool for each domain was assessed by inspecting the ratio of the first eigenvalue to the second eigenvalue using scree plots. Domains with ratios greater than or equal to 3 to 1 were considered sufficiently unidimensional to relate to a single construct (Ackerman, 1989; Reckase, 1979; Reise, Cook, & Moore, 2014; Reise & Waller, 2009).

#### Approaches 2 and 3

In order to fully examine the properties of the E‐SWAN, we included two additional modeling approaches; (a) modeling all questions for each domain together in a bifactor model and; (b) modeling all scales separately (i.e., separate models for unidirectional and E‐SWAN scales). Detailed descriptions of these additional approaches, and results, can be found in the Supporting Information.

#### ROC curves for diagnostic prediction

To determine the ability of E‐SWAN scales to predict DSM‐based diagnoses in the CMI‐HBN sample, and their comparability to traditional scales, we generated Receiver Operating Characteristic (ROC) curves for all scales using clinician consensus diagnoses from the KSADS (Kaufman et al., 1997). We then calculated and compared Area Under the Curve (AUC) between E‐SWAN scales and unidirectional scales measuring the same disorder.

All analyses were carried out in R (Chalmers, 2012; Gamer, Lemon, & Fellows, 2007; Koenker, 2013; Revelle, 2011; Rizopoulos, 2006; Robin et al., 2011; Rosseel, 2012; Van der Ark, 2007). All analyses were performed respecting the categorical nature of the items.

## Results

### Distributional properties

**Figure 2:**
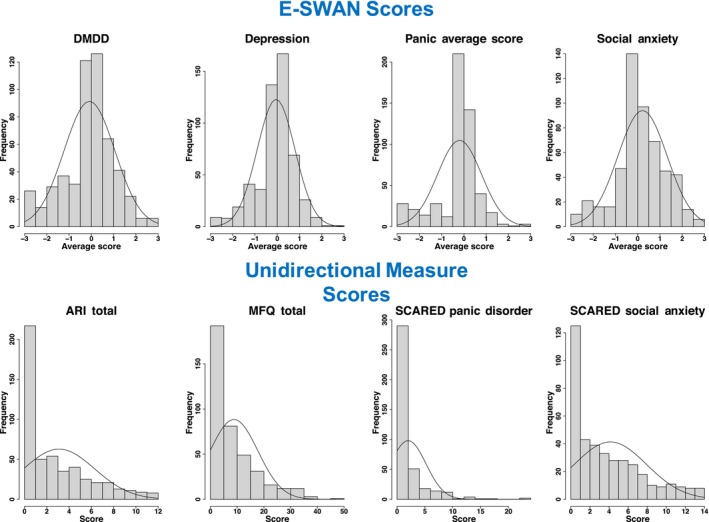
Distribution of E‐SWAN Scores and unidirectional Measure Scores. Distribution of scores from E‐SWAN scales, shown in the top panel, compared with the distribution of scores from their unidirectional counterparts, shown in the bottom panel [Mood and Feelings Questionnaire (MFQ), Screen for Child Anxiety and Related Disorders (SCARED), Affective Reactivity Index (ARI)] [Colour figure can be viewed at http://www.wileyonlinelibrary.com]

Consistent with the SWAN, all E‐SWAN scales in the CMI‐HBN sample were approximately symmetrically distributed, in contrast to their counterparts (Figure 2, Table 1). A sample of reports from 250 parents and 250 self‐reports in the PA sample yielded similar distributional properties, confirming that these findings were not specific to CMI‐HBN (Figure S2). The mean E‐SWAN scores for the PA samples were lower than the CMI‐HBN sample, indicating a higher rating of strengths in the PA sample. *t* tests show that the difference in sample means is statistically significant across all domains (Table S1). Cronbach's alpha showed high internal consistency of items within each of the E‐SWAN scales (*α* .91–.98; Table 1). When comparing scores between the E‐SWAN and unipolar counterpart scales, Cronbach's alpha and Pearson correlation coefficients showed moderate levels of internal consistency (values > .5) and correlation, respectively (Table 1). To examine the added benefit of the bidirectional scales, we split the E‐SWAN ratings into those that scored at or above 0 (reflecting a degree of psychopathology as in traditional scales) and those that scored below 0 (higher levels of strengths). We again calculated Cronbach's alpha, and Pearson correlations on the two groups. As expected, we found that internal consistency and correlations were markedly lower among those that scored below 0 (i.e., the strengths group). This indicates that the unidirectional measures no longer align with the E‐SWAN scales when measuring positive behaviors rather than symptoms. This relationship was consistently seen across all domains assessed by the E‐SWAN (Table 1). Quantile regression showed that the traditional scales vary as a function of the E‐SWAN scores. Stronger correlations are seen between the traditional scales and the E‐SWAN scales at the extreme (pathologic) end of the trait (Figure S3).

**Table 1:** Descriptive statistics and measures of concordance and reliability

		Individual scales									Interscale relationship	
		Mean	Median	Mode	Range	Skewness	Kurtosis	Variance	Omega	Cronbach Alpha	Cronbach Alpha	Pearson Correlation
Major Depression	E‐SWAN Depression	0.08	−0.05	0	−3, 3	−0.73	1.51	0.71	.85	.91	.66	.49
	MFQ	8.80	6.00	0	0, 47	1.39	1.57	80.59	.93	.92		
	MDD (E‐SWAN ≥ 0)										.73	.56
	MDD (E‐SWAN < 0)										.37	.23
Social Anxiety	E‐SWAN Social Anxiety	0.17	0.22	0	−3, 3	−0.36	0.59	1.21	.85	.922	.65	.49
	SCARED Social	4.14	3.00	0	0, 14	0.85	−0.15	14.5	.85	.88		
	Social Anxiety (E‐SWAN ≥ 0)										.66	.51
	Social Anxiety (E‐SWAN < 0)										.25	.14
Panic Disorder	E‐SWAN Panic	0.00	−0.20	0	−3, 3	−1.19	2.05	1.01	.93	.97	.54	.36
	SCARED Panic	1.98	1.00	0	0, 23	2.96	11.86	10.32	.87	.85		
	Panic Disorder (E‐SWAN ≥ 0)										.75	.56
	Panic Disorder (E‐SWAN < 0)										.29	.17
DMDD	E‐SWAN DMDD	0.03	−0.09	0	−3, 3	−0.43	0.39	1.32	.97	.98	.75	.59
	ARI	0.21	0.16	0	0, 12	0.97	0.04	10.2	.92	.91		
	DMDD (E‐SWAN ≥ 0)										.67	.51
	DMDD (E‐SWAN < 0)										.54	.36

Table shows descriptive statistics for each E‐SWAN scale and unidirectional counterparts [Mood and Feelings Questionnaire (MFQ), Screen for Child Anxiety and Related Disorders (SCARED), Affective Reactivity Index (ARI)]. Measures of reliability are shown between each E‐SWAN scale and its unidirectional counterparts for all participants, and for participants grouped by E‐SWAN score. To demonstrate the added value of the E‐SWAN, the data was split into two groups, one that included participants that scored at or above 0 on the E‐SWAN scale (those with strengths in the given domain) and those that scored below 0 (those with weaknesses, or symptoms in the given domain).

#### Psychometric approach 1

**Figure 3:**
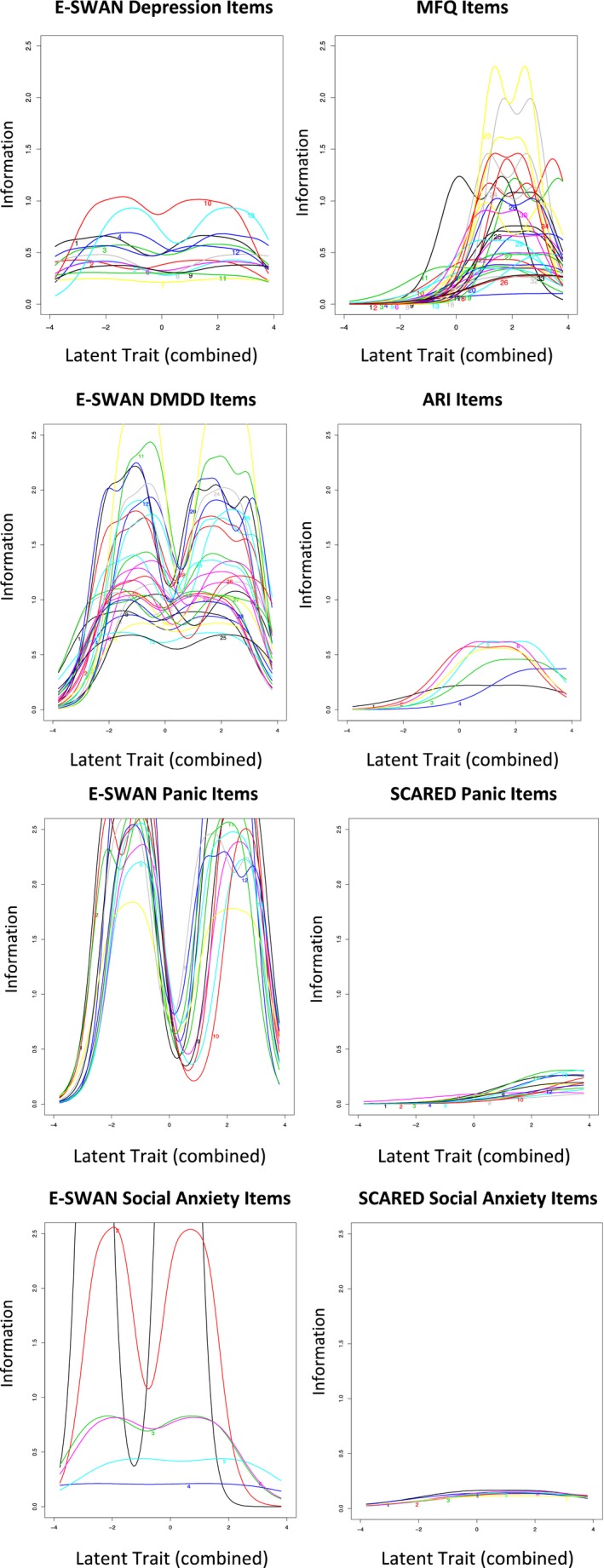
Item Information Curves for Unidimensional IRT with jointly modeled scales. Plots show the area along the latent trait for which each item is capturing information. For each domain, all E‐SWAN items capture information across the entire latent trait (strengths and weaknesses), whereas items on the unidirectional scales [Mood and Feelings Questionnaire (MFQ), Screen for Child Anxiety and Related Disorders (SCARED), Affective Reactivity Index (ARI)], with very few exceptions, only capture information at or above the mean (weaknesses) [Colour figure can be viewed at http://www.wileyonlinelibrary.com]

All questions for a given domain analyzed together as a single unidimensional IRT model. A prerequisite for this unidimensional IRT analyses is the demonstration that the data being analyzed meet assumptions of sufficient unidimensionality. As can be seen from the scree plots (Figure S4), there was a substantial difference between the first and second eigenvalues for all the composite item pools. All item pools had eigenvalue ratios greater than 3 to 1, providing support for the assumption that those scales are measuring, at least partially, the same latent trait (Figure S4). The IRT models showed that the E‐SWAN items are capturing information from *z*‐scores of −3 to +3 along the latent trait, while the traditionally developed measures only capture information from *z*‐scores >0 along the latent trait. As an example, in Figure 3, when E‐SWAN‐Depression and MFQ items were included in the same IRT model, we can see that all E‐SWAN‐Depression items are informative across the entire latent trait, whereas MFQ items, with a few exceptions, only inform 1 SD above the mean.

#### Psychometric approaches 2 and 3

Both sets of analyses showed similar results to approach one. See Appendix S1, Tables S2–S17, Figures S5–S7 for more information.

#### Predictive value for DSM diagnosis

**Figure 4:**
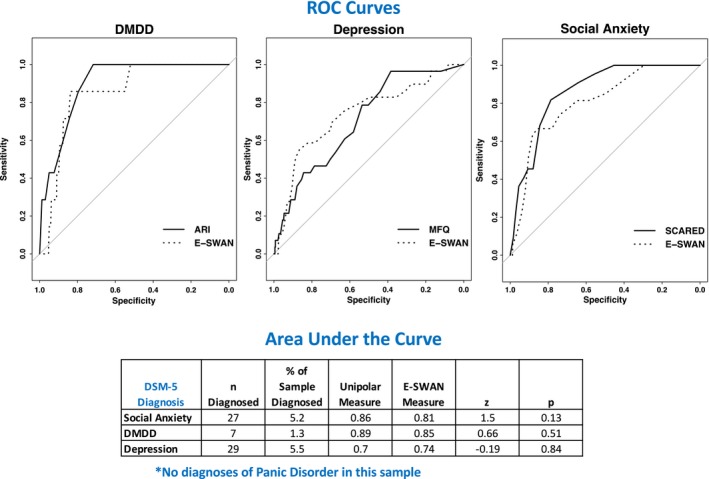
ROC Curves. Receiver Operating Characteristic curves representing diagnostic capabilities of both E‐SWAN and unidirectional scales for DMDD, Depression, and Social Anxiety [Mood and Feelings Questionnaire (MFQ), Screen for Child Anxiety and Related Disorders (SCARED), Affective Reactivity Index (ARI)] [Colour figure can be viewed at http://www.wileyonlinelibrary.com]

We generated ROC curves for all scales using diagnoses generated from the K‐SADS (Figure 4). Both E‐SWAN and traditional scales performed well (AUC values 0.7–0.89), indicating that they are comparable screening tools and giving increased support for the validity of the E‐SWAN questionnaires. As depicted in Figure S8, we found that predictive value of each E‐SWAN scale was specific to the disorder that it was intended to measure. The E‐SWAN is capturing not only the absence of psychopathology but also the presence of positive traits; this means that a child in the pathologic range for one trait may have strengths in another, which could be informative for a clinician (Figures S9 and S10).

## Discussion

Inspired by the SWAN, we developed and tested a generalized framework for constructing questionnaires to assess the full range of behavior underlying DSM symptoms, when considered as an endpoint of a dimension. When compared to the unidirectional scales, the E‐SWAN scales exhibited distributional properties that were symmetric rather than highly skewed or truncated. As predicted, for each trait, a strong correspondence was noted between the E‐SWAN scores and traditional scale scores among individuals at the high (pathological) end, but not at the low end. IRT analyses suggested that in contrast to traditional scales, the E‐SWAN subscales exhibited good discrimination and reliability across the full latent trait (*z*‐scores from −3 to +3; reliabilities ranging from 0.77 to 0.97) – not just at the high end as unidirectional scales. Finally, we demonstrated the ability to generate both parent and self‐report questionnaires using the E‐SWAN framework. Consistent with the data from CMI‐HBN participants, our online sample from PA yielded a symmetric distribution, although shifted slightly to the left (i.e., less symptomatic), as would be expected given the differences in recruitment strategies.

The ability to meaningfully and reliably account for variance across the entirety of a population becomes more important as biological and epidemiologic studies shift away from categorical, syndromic characterizations of psychiatric illness. Efforts such as the NIMH Research Domain Criteria Project have successfully drawn attention to the potential added value of dimensional characterizations that cut across the diagnostic boundaries specified by DSM and ICD (Insel et al., 2010). Yet the vast majority of questionnaires focused on mental health are limited in their ability to characterize variation among individuals beyond the symptomatic segment of the population (Axelrud et al., 2017; Greven et al., 2018). The E‐SWAN framework offers a viable alternative for improving our ability to differentiate individuals that are nonsymptomatic in a given domain. It does so without losing track of the clinical significance of identifying a pathological range of the trait (which is used as the departure point for characterizing the trait). The data from the E‐SWAN are more statistically appropriate for dimensional analysis. ROC analyses highlight potential comparability of the E‐SWAN for the detection of illness, while having an increased ability to detect strengths. However, we do not take this to suggest superiority of the E‐SWAN at detecting illness; prior to using the E‐SWAN for this purpose, large scale data sets would need to be collected to establish proper cutoffs.

While the present work used the total score obtained for a given scale as the unit of analysis, future work may benefit from consideration of individual item scores. Similar to overall questionnaire scores, individual items of the E‐SWAN are intended to represent a bidirectional dimension. This property can be particularly valuable for efforts focused on the identification of abilities that may have protective effects or confer resilience, as well as disabilities associated with impairment. For example, when evaluating individuals with equivalent symptom profiles, though differing outcomes, the presence or absence of strengths may result in different outcomes. The E‐SWAN scales can be used to capture such distinctions. It is important to note that ability to measure strengths and weaknesses along a single continuum does not mean necessarily that the underlying biology is unidimensional. Brain–behavior relationships can differ across the range of a dimension (i.e., one biological process can contribute more heavily toward weaknesses and another toward strengths); the neuroimaging literature already has examples of this when we think of the age‐related changes in the neural correlates of constructs such as intelligence and reading abilities (Koyama et al., 2011; Shaw et al., 2006). It is important to acknowledge that the conceptualization of strengths in mental health is an ongoing point of discussion. In the present work, we defined strengths as the high end of abilities relevant to DSM‐5 criteria. In most cases, one could readily identify the relevance of the construct at the strengths end (e.g., ‘limit feelings of sadness’, ‘enjoy activities’, ‘maintain appropriate sleep’ and ‘maintain appropriate appetite’). Though, this is not always true – particularly, for the physiologic‐related abilities identified in Panic Disorder (e.g., ‘Maintain a consistent heart rate’). Future work will focus on the identification of additional strengths that can bring us toward high levels of wellness and function, which would not be readily discerned from the E‐SWAN framework.

There are a number of limitations of the E‐SWAN framework that suggest areas for improvement. First, a key assumption – that the underlying dimension of behavior described is bidirectional and symmetrically distributed in the general population – may not hold for all disorders. While likely reasonable for most DSM disorders, some, such as PTSD and Substance Use Disorders, represent clear instances where only a subset of the population has had exposure to trauma or a given substance (American Psychiatric Association, 2013). For these disorders, the prompts and range of responses can be changed to create a distribution in a subset of the population defined by the presence of a particular exposure (e.g., stressor, substance use). Questionnaires for both of these disorders are under development (see eswan.org for current drafts). Second is the potential for biased reporting, which can arise from either a skewed perception of ‘other children the same age’ on the part of the informant, or a bias to see a child as more (or less) able than they are (e.g., the ‘Lake Wobegon Effect’; Phillips, 1990). Arguably such biases are also present in unidirectional questionnaires, though centered more around ratings of frequency. The collection of data from multiple informants, such as teachers, is a common approach to overcoming such biases. Third, when developing questionnaires, we focused on clarity, precision, and general applicability of questions, and tested the readability of questions using online software, however, the questions could benefit from more formal and extensive user testing of readability, acceptability, and comprehensibility. This testing could help to further refine the questions. Fourth, with the current data available, we do not have a measure of test–retest reliability. To further validate the E‐SWAN, future studies should focus on collecting and analyzing test–retest data. Finally, it would be of value to compare the E‐SWAN scales against other dimensional measures and positive measures, however, this is not feasible with the data currently available. Future studies can collect data to allow for this analysis.

As demonstrated in our comparison of results from CMI‐HBN and PA, the mean of the distribution obtained in a given sample can vary depending on the specific segment of the population sampled. Within a given study, this is not necessarily a problem. However, if not considered, such variation can lead to confounds or biases when attempting to compare or combine across studies, or to develop clinical cutoffs. An effective solution may be the generation of appropriately sized normative samples to serve as a reference. This need is not different from any other questionnaire. A positive aspect of the E‐SWAN versus other questionnaires is that it can be used to compare distributional characteristics to understand differences among samples in their entirety.

It is worth noting a few potential misinterpretations of the E‐SWAN framework that we believe should be avoided. First, the probes are not intended suggest that ‘the average’ should be viewed as an ‘ideal’ or target. Instead, we are using the average as a logical reference point (also used in the DSM approach to anchor symptoms) that allows raters to consider a given ability in the context of the population, with the added information related to direction of an extreme strength as well as an extreme weakness. Thus, the average is being used as a reference for measurement, not a standard to aim for. Second, it is important to note that some ‘strengths’ at an extreme or in isolation can actually be deleterious, at least in some contexts. Arguably, it is the combination of measured strengths and weaknesses that is most informative regarding the status of a child. We believe properties of the E‐SWAN allow for just that; researchers can look for multivariate profiles that engender higher or lower function, rather than just a given strength in isolation, which can be a plus or minus depending on the larger context. Third, given that E‐SWAN items are directly related to the content of DSM 5 criteria, the framework is not all inclusive with respect to strengths and weaknesses that may be relevant to psychiatric illness. However, we do believe that the using the DSM 5 criteria to define content does ensure the relevance of this dimensional approach for evaluation of psychiatric disorders. Fourth, a higher number of response options and a higher number of items might increase the amount of information from one instrument if compared to another; therefore, the amount of information provided by both instruments is not necessarily comparable. However, our main interest in this analysis is not to compare the amount of information from each scale, but to investigate the area of the latent trait from which each instrument provides information. We hypothesized (and documented) that E‐SWAN scales capture information about the full latent trait, whereas the legacy measures only capture information from one end of the latent trait, from individuals with a high level of symptoms.

In the spirit of collaboration and open science, all E‐SWAN questionnaires are freely available for use and can be accessed at http://www.eswan.org, licensed Creative Commons (CC) BY 4.0 to encourage maximal dissemination and application of the questionnaires. It is our hope that other investigative teams will join in the effort to create the full range of E‐SWAN questionnaires encompassing all major psychiatric syndromes.

**Key points**

- Myriad questionnaires are available for measuring psychiatric illness dimensionally. However, the vast majority are based on detection of the presence of problematic behaviors and symptoms.
- The Strengths and Weaknesses Assessment of ADHD Symptoms and Normal Behavior (SWAN) provides a potentially valuable model for bidirectional questionnaire design.
- The methodology of the SWAN was extended for the E‐SWAN to address four additional DSM‐5 disorders; Depression, Social Anxiety, Panic Disorder, and DMDD.
- The E‐SWAN bidirectional scales can capture the full spectrum of the population distribution of behavior underlying these DSM disorders.
- The additional information provided by the E‐SWAN can better inform examination of inter‐individual variation in population studies, as well as facilitate the identification of factors related to resiliency in clinical samples.

## Supporting information


**Appendix S1.** Modeling and results of psychometric approaches 2 and 3.
**Table S1.** *t* test results comparing mean scores between CMI‐HBN and prolific academic samples.
**Tables S2–S17.** More information on psychometric approaches 2 and 3.
**Figure S1.** E‐SWAN items.
**Figure S2.** Prolific academic parent report and HBN parent report.
**Figure S3.** Quantile regression plots.
**Figure S4.** Scree plots for jointly modeled scales.
**Figures S5–S7.** More information on psychometric approaches 2 and 3.
**Figure S8.** ROC curves for all scales.
**Figure S9.** Distribution of E‐SWAN scores by diagnosis.
**Figure S10.** Distribution of SDQ prosocial scores by E‐SWAN score quintile.
