# Supplementary material for: Measuring strengths and weaknesses in dimensional psychiatry
Source: J Child Psychol Psychiatry. 2019 Aug 18;61(1):40–50. doi: 10.1111/jcpp.13104 (PMC6916607; doi:10.1111/jcpp.13104)
Supplement: Supplementary file 1 — Appendix S1. Modeling and results of psychometric approaches 2 and 3. Table S1. t test results comparing mean scores between CMI‐HBN and prolific academic samples. Tables S2–S17. More information on psychometric approaches 2 and 3. Figure S1. E‐SWAN items. Figure S2. Prolific academic parent report and HBN parent report. Figure S3. Quantile regression plots. Figure S4. Scree plots for jointly modeled scales. Figures S5–S7. More information on psychometric approaches 2 and 3. Figure S8. ROC curves for all scales. Figure S9. Distribution of E‐SWAN scores by diagnosis. Figure S10. Distribution of SDQ prosocial scores by E‐SWAN score quintile. [file JCPP-61-40-s001.docx]

**Supporting information -- Measuring strengths and weaknesses in dimensional psychiatry – by Alexander *et al*.**

**Table of Contents**

***Supporting information*** ***1***

**Appendix S1. Modelling and results of psychometric approaches 2 and 3.** **2**

**Figure S1.** **E-SWAN Items.** **4**

**Figure S2.** **Prolific Academic Parent Report and HBN Parent Report.** **5**

**Table S1.** **T-Test Results Comparing Mean Scores Between CMI-HBN and Prolific Academic Samples.** …………………….**6**

**Figure S3.** **Quantile Regression Plots** **6**

**Figure S4. Scree plots for jointly modeled scales.** **7**

**Table S2. Proportion Variance for Bifactor Models.** **7**

**Table S3. Discrimination (α) and difficulty (β) parameters for E-SWAN Depression and Mood and**

**Feelings Questionnaire (MFQ)** **8**

**Table S4. Discrimination (α) and difficulty (β) parameters for E-SWAN Social Anxiety and Screen for**

**Child Anxiety and Related Disorders (SCARED) Social Anxiety** **9**

**Table S5. Discrimination (α) and difficulty (β) parameters for E-SWAN Panic Disorder and Screen for**

**Child Anxiety and Related Disorders (SCARED) Panic Disorder** **10**

**Table S6. Discrimination (α) and difficulty (β) parameters for E-SWAN DMDD and Affective Reactivity**

**Index (ARI)** **11**

**Figure S5. Test Information Function Plots.** **12**

**Figure S6. Item Information Curves** **13**

**Figure S7. Sample Item Characteristic Curves.** **14**

**Figure S8. ROC Curves for All Scales.** **15**

**Figure S9. Distribution of E-SWAN Scores by Diagnosis** **16**

**Figure S10. Distribution of SDQ Prosocial scores by E-SWAN score quintile.** **17**

**Table S7. Measures of Fit for Confirmatory Factor Analysis.** **18**

**Table S8. Monotonicity results** **18**

**Table S9. Item Response Theory Parameters for E-SWAN Depression Scale.** **21**

**Table S10. Item Response Theory Parameters for E-SWAN Panic Disorder Scale.** **21**

**Table S11. Item Response Theory Parameters for E-SWAN Social Anxiety Scale.** **22**

**Table S12. Item Response Theory Parameters for E-SWAN DMDD Scale.** **23**

**Table S13. Item Response Theory Parameters for Affective Reactivity Index (ARI).** **24**

**Table S14. Item Response Theory Parameters for Mood and Feelings Questionnaire (MFQ).** **25**

**Table** **S15. Item Response Theory Parameters for Screen for Child Anxiety and Related Disorders**

**(SCARED) Panic Disorder.** **26**

1

**Table S16. Item Response Theory Parameters for Screen for Child Anxiety and Related Disorders**

**(SCARED) Social Anxiety.** **27**

**Table S17. Measurement Invariance between HBN and PA sample on Social Anxiety and Depression**

**E-SWAN Scales** **27**

**Appendix S1.** Modelling and results of psychometric approaches 2 and 3.

***Modeling Approach 2*.** Bifactor models with bidirectional and unidirectional scales modeled as a common trait and specific dimensions at the same time. This analysis aims to address the limitations of ignoring slight differences in the latent trait measured by each unidirectional and bidirectional scales from the former analysis.

***Results of Modeling Approach 2 – Bifactor Item Response Theory Models*.** A similar pattern is seen between the unidimensional models and the bifactor models. As seen in Supplemental Table 1, the proportion of variance, which is due to a common factor, is higher for the E-SWAN and Unipolar scales modeled together, than for either scale independently. The discrimination and difficulty parameters in a model, which models the common variance (i.e., variance which is common to both instruments) and specific variance (i.e., variance which is specific to each instrument) can be found in Supplemental Tables 2-5.

***Modeling Approach 3*.** Two scales for each domain separately in a unidimensional IRT model. This analysis provides item level psychometric information for each scale separately. Confirmatory Factor Analysis was used to evaluate the model’s goodness of fit to the data. Additionally, we analyzed the E-SWAN questions with the HBN and PA samples combined, and calculated the measurement invariance, to determine whether the same trait was being measured in each sample.

For unidimensional and multidimensional IRT analyses, we used Graded Response Models (GRM) (Samejima 1968) to calculate each item’s discrimination parameter, which indexes the strength of the relationship between each item and the latent trait, and each item’s difficulty parameter, which indexes in each interval of the latent trait where the item provides information (Baker 2001). Test Information Function (TIF) curves were plotted for each instrument. These plots depict how well the overall test discriminates individuals, and the precision of the measurement at various levels of the latent trait. Similarly, Item Information Function (IIF) curves show how each individual item on the questionnaire performs. Finally, Item Characteristic Curves (ICC) plots depict the difficulty parameter, which represents the probability of a respondent endorsing each of the response options (e.g., far above average to far below average) along the latent trait for each instrument.

2

Confirmatory Factor Analysis (CFA) models were also used for questionnaires modeled separately to investigate goodness of fit. These models used mean and variance adjusted weighted least squares (WLSMV) estimators, which account for the ordinal nature of the data. From this we assessed model fit using the Comparative Fit Index (CFI), Tucker-Lewis Index (TLI) and Root Mean Square Error of Approximation (RMSEA). CFI and TLI values ≥0.90 represent an acceptable fit, and ≥0.95 a good fit. Values of the RMSEA≤0.08 represent an acceptable fit, and ≤0.05 a good fit(Hu & Bentler 1999; Brown 2006). In addition, we tested the assumption of monotonicity for the E-SWAN items.

***Results of Modeling Approach 3*** *-* ***Modeling the two scales for each domain separately in a unidimensional model*.** Supplemental Figure 5 shows the Test Information Function curves and reliability for each scale. As can be seen in this figure, the E-SWAN scales provide reliable information from -3 to 3 standard units above the mean (reliability values 0.77-0.97 [Supplementary Tables 8-15]). The unidirectional scales only capture reliable information from 0 to 3 standard units above the mean of the latent trait. CFA results for scales modeled independently are shown in Supplementary Table 6. Some models were adjusted residual correlations between items of similar content. All models showed good fit based on CFI and TLI (all values above 0.9), and most showed acceptable to good fit for RMSEA (all values <= 0.08) (Supplementary Table 6). The majority of E-SWAN items showed monotonicity, indicating that higher response categories are associated with higher levels of the latent trait (Supplementary Table 7).

The Item Information Curves in Supplementary Figure 6 show that this same relationship exists for the individual items that make up the E-SWAN and unidirectional questionnaires. The E-SWAN questions capture information across the full latent trait, while the unidirectional scales only capture information at the high end of the latent trait.

Supplementary Figure 7 shows an example of the Item Characteristic Curve for one question on the E-SWAN Depression subscale and one question on the MFQ. These curves show the probability of a particular answer choice being endorsed at each level of the latent trait. Supplementary Tables 8-15 indicate at which point along the latent trait there is a 50% probability of transitioning to the next response choice.

Models with the combined responses from the HBN and PA samples showed measurement invariance ( CFI values < 0.01 and RMSEA values < 0.015), indicating that the same latent trait is being measured in both samples (Supplementary Table 16).

3

**Figure S1.** E-SWAN Items.

Four items from each E-SWAN scale are compared side by side with the DSM criteria from which they were developed


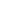

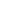

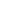

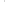


|  | **DSM Criteria** | **E-SWAN Question** | |  |
| --- | --- | --- | --- | --- |
|  |  | **Compared to other childre of the same age, in recent weeks, how well does this child:** | |  |
|  | A. Marked fear or anxiety about one or more social situations in which the individual is exposed to | 1. Tolerate feelings of anxiety in social situations | |  |
|  | possible scrutiny by others |  |  |  |
|  |  |  |  |  |
| **Social** | B. The individual fears that he or she will act in a way that will show anxiety symptoms that will be | 2. Handle fears of appearing anxious in social situations | |  |
|  | negatively evaluated (i.e., will be humiliating or embarassing, or will lead to rejection by others) |  |  |  |
| **Anxiety** |  |  |  |  |
|  |  |  |  |  |
|  | C. The social situations almost always provoke anxiety | 3. Stay relaxed in social situations | |  |
|  | D. The social situations are avoided or endured with intense fear or anxiety | 4a. | Seek out social activities |  |
|  |  | 4b. | Enjoy participating in social activities |  |
|  |  |  |  |  |
|  | 1. Depressed mood most of the day, mearly every day, as indicated by either subjective report or | 1A. Limit feelings of sadness | |  |
|  | observation made by others (Note: in children/adolescents can be irritable mood) | 1B. Limit feelings of irritability | |  |
| **Depression** | 2. Markedly diminished interest or pleasure in all, or almost all, activites most of the day, nearly | 2A. Show interest in preforming activities | |  |
|  | every day (as indicated by either subjective account or observation) | 2B. Enjoy activities | |  |
|  | 3. Significant weight loss when not dieting or weight gain (e.g., a change of more than 5% of body | 3A. Maintain stable weight | |  |
|  | weight ina month), or decrease or increase in appetite nearly every day | 3B. Maintain appropriate appetite | |  |
|  | 4. Insomnia or hypersomnia nearly every day | 4. Sleep for an appropriate amount of time | |  |
|  | 1. Palpatations, pounding heart, or accelerated heart rate | 1. Keep a regular heartbeat | |  |
| **Panic** | 2. Sweating | 2. Keep dry and cool | |  |
| **Disorder** | 3. Trembling or shaking | 3. Keep hands steady | |  |
|  | 4. Sensations of shortness of breath or smothering | 4. Breath at their typical speed | |  |
|  |  | 1a. | Avoid or control ‘arguing or yelling’ at home |  |
|  |  | 1b. | Avoid or control ‘arguing or yelling’ with friends |  |
|  |  | 1c. Avoid or control ‘arguing or yelling’ at school | |  |
|  | A. Severe recurrent temper outbursts manifested verbally and/or behaviorally that are grossly out of | 2a. | Avoid or control ‘getting into fights’ at home |  |
|  |  | 2b. | Avoid or control ‘getting into fights’ with friends |  |
|  | proportion in intensity or duration to the situation or provocation |  |  |  |
| **DMDD** |  | 2c. Avoid or control ‘getting into fights’ at school | |  |
|  |  |  |  |  |
|  |  | 3a. | Avoid or control ‘taking anger out on objects or throwing things at people’ at home |  |
|  |  |  |  |  |
|  |  | 3b. | Avoid or control ‘taking anger out on objects or throwing things at people’ with friends |  |
|  |  | 3c. Avoid or control ‘taking anger out on objects or throwing things at people’ at school | |  |
|  | D. The mood between temper outbursts is persistently irritable or angry most of the day, nearly | 9a. | Exhibit a good mood throughout the day, most days of the week at home |  |
|  |  | 9b. | Exhibit a good mood throughout the day, most days of the week with friends |  |
|  | every day, and is observable by others |  |  |  |
|  |  | 9c. Exhibit a good mood throughout the day, most days of the week at school | |  |
|  |  |  |  |  |

4

**Figure S2.** Prolific Academic Parent Report and HBN Parent Report.

Plots comparing distribution of E-SWAN scores from HBN parent

reports and online parent reports gathered through prolific academic. Both samples show similar distributions, with the online sample having a slightly lower mean. The tables to the right provide the skewness and kurtosis for the samples obtained online.

| **Parent Report** |  | **Parent Report** | | |  |  |
| --- | --- | --- | --- | --- | --- | --- |
|  |  |  | **Mean** | **SD** | **Skewness** | **Kurtosis** |
|  | **Depression Average Score** |  | -0.64 | 0.76 | -0.25 | 3.19 |
|  | **DMDD Average Score** |  | -0.76 | 0.94 | -0.22 | 2.53 |
|  | **Social Anxiety Average Score** | | -0.34 | 1.1 | -0.09 | 3.41 |
|  | **Panic Average Score** |  | -0.71 | 0.94 | -0.57 | 2.77 |


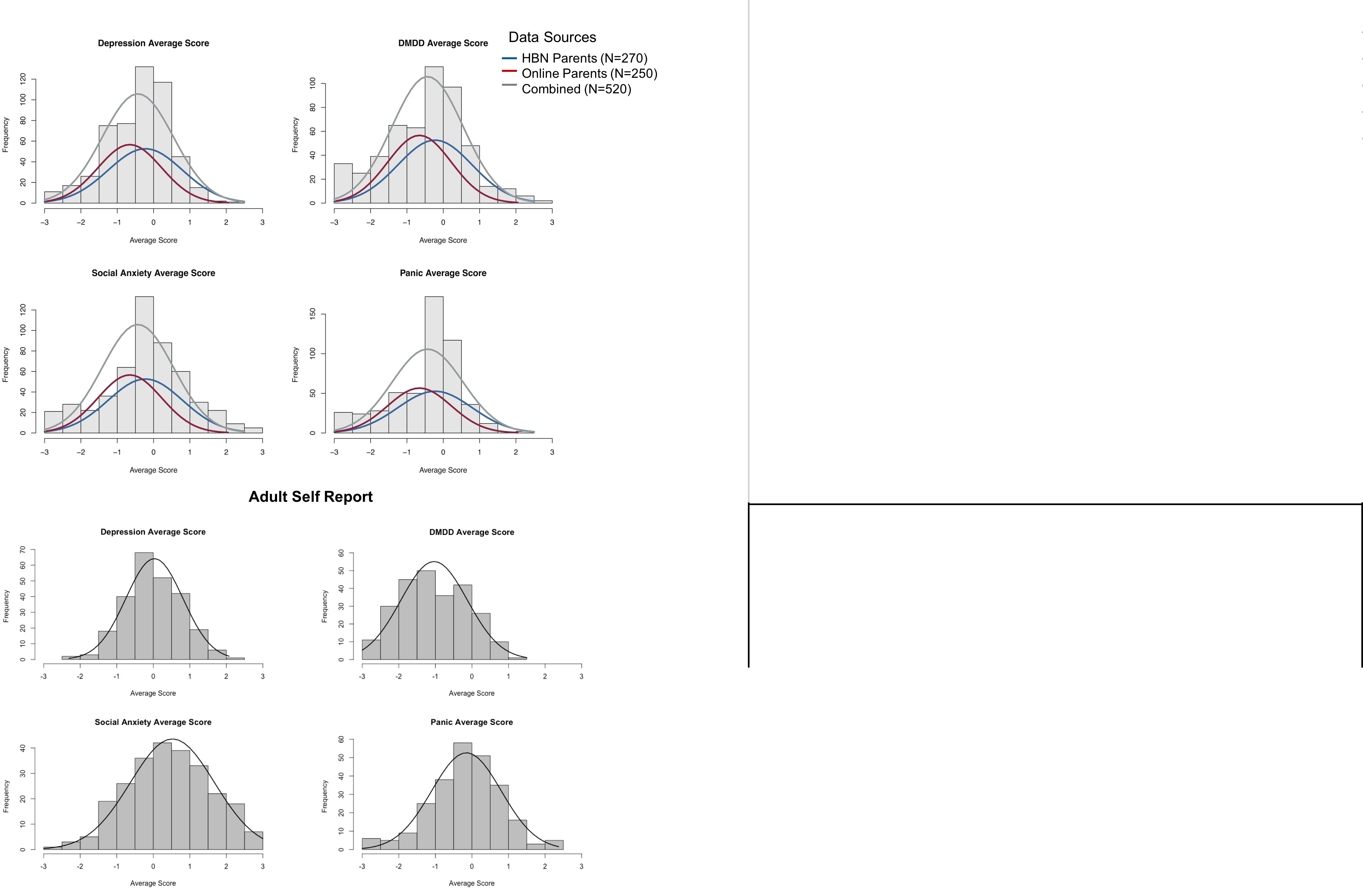


**Adult Self Report**

|  | **Mean** | **SD** | **Skewness** | **Kurtosis** |
| --- | --- | --- | --- | --- |
| **Depression Average Score** | 0.03 | 0.77 | 0.06 | 2.79 |
| **DMDD Average Score** | -1.03 | 0.91 | 0.12 | 2.25 |
| **Social Anxiety Average Score** | 0.52 | 1.1 | -0.05 | 2.63 |
| **Panic Average Score** | -0.14 | 0.98 | -0.24 | 3.55 |

5

**Table S1.** T-Test Results Comparing Mean Scores Between CMI-HBN and Prolific Academic Samples.

The table shows that the mean scores for the Prolific Academic (PA) sample are significantly higher than the CMI-HBN sample, indicating that the PA sample endorsed more strengths than the CMI-HBN Sample.


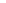

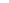

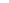

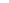

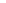


|  | **CMI-HBN** | **PA** | **p-value** |
| --- | --- | --- | --- |
| **DMDD** | -0.085 | -0.767 | 2.20E-16 |
| **Major Depression** | -0.052 | -0.641 | 2.20E-16 |
| **Panic Disorder** | -0.196 | -0.705 | 1.59E-11 |
| **Social Anxiety** | 0.218 | -0.341 | 1.56E-10 |

**Figure S3.** Quantile Regression Plots*.*

Quantile regression plots showing that the traditional scales vary as a function of the E-SWAN scores. These plots show that the relationship between traditional scale scores and E-SWAN scores differ based on the strength of the disorder trait. Stronger correlations are seen between the traditional scales and the E-SWAN scales at the extreme (pathologic) end of the trait and lower correlations are seen at the low (strengths) end of the trait. This indicates that the two scales are obtaining different information at the lower (strengths) end of the trait.


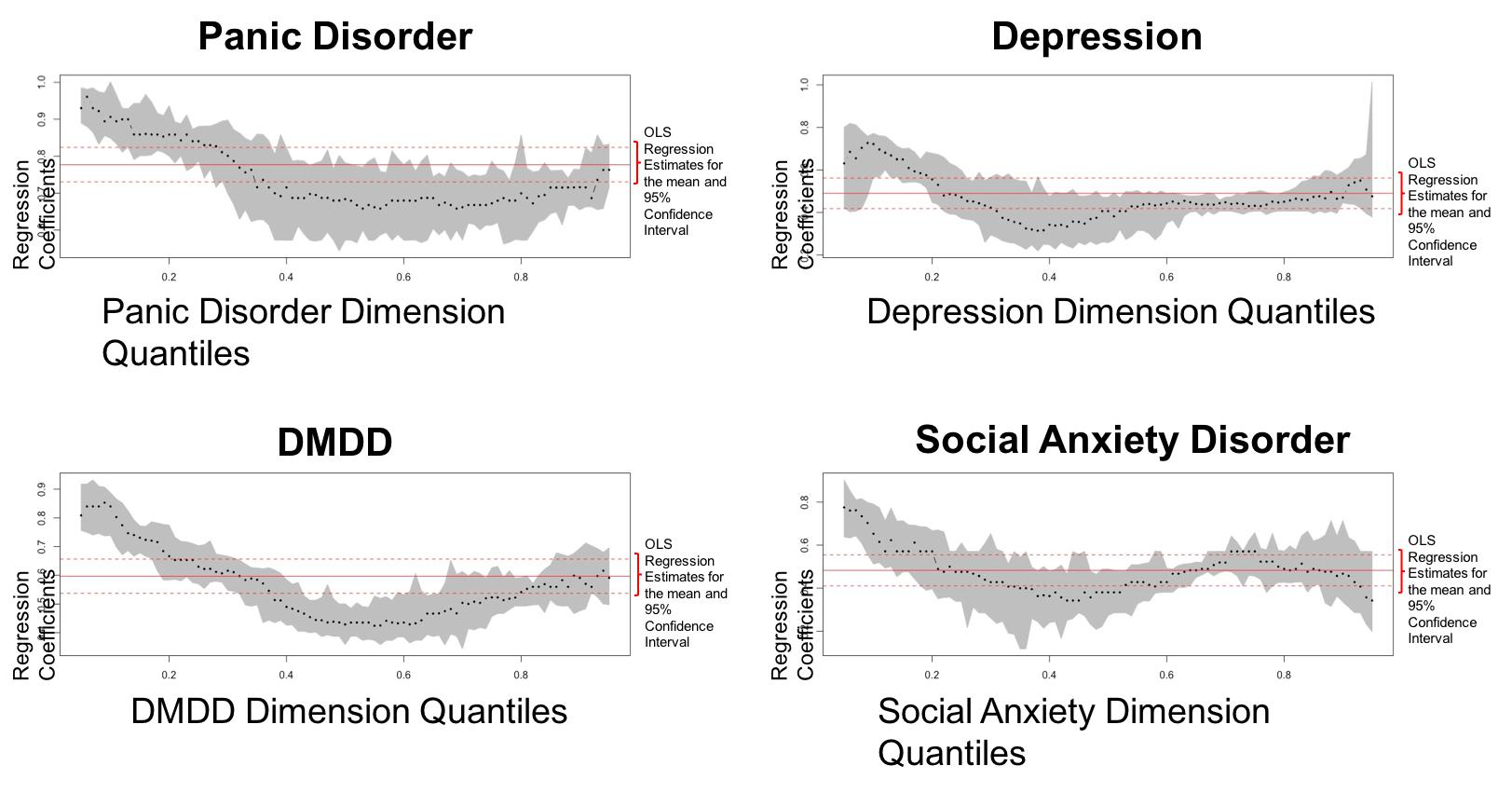


6

**Figure S4.** Scree plots for jointly modeled scales.

Scree plots show unidimensionality when scales are modeled together, indicating that both scales are measuring one latent trait.


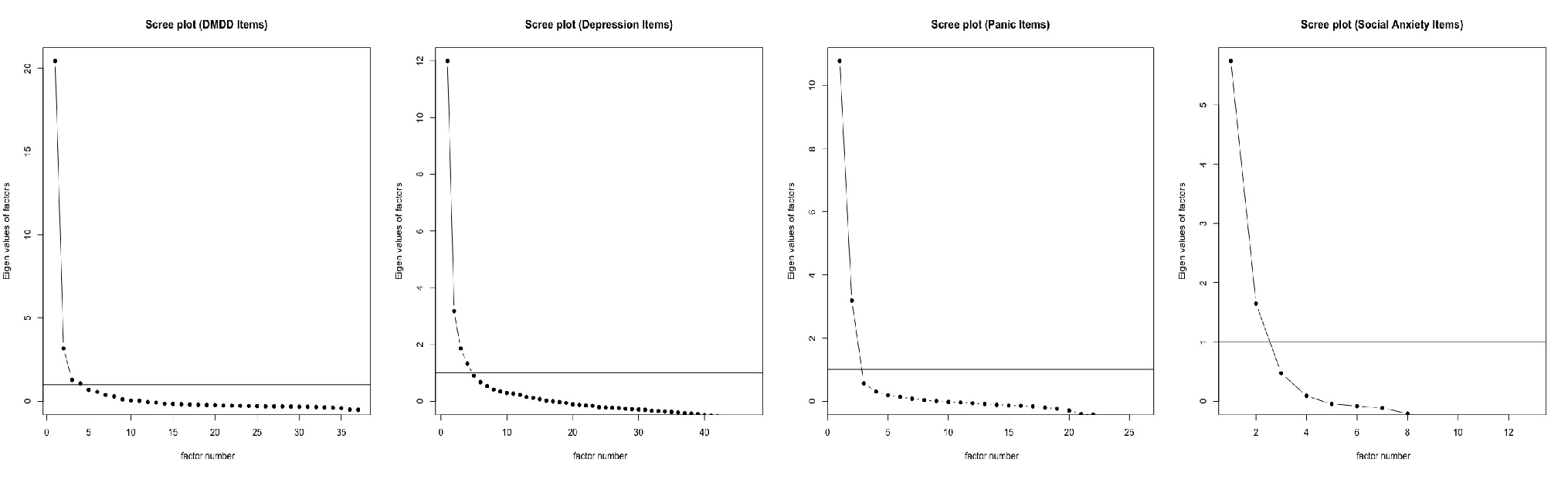


|  | **First** | **Second** |  |
| --- | --- | --- | --- |
|  | **Eigenvalue** | **Eigenvalue** | **Ratio** |
| **DMDD** | 20.4 | 3.2 | 6.5 |
| **Depression** | 12 | 3.2 | 3.75 |
| **Panic Disorder** | 10.8 | 1.6 | 3.5 |
| **Social Anxiety** | 5.7 | 1.6 | 3.5 |

**Table S2.** Proportion Variance for Bifactor Models.


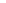

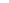

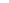

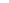

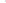


|  |  |  | **Specific aspects of** |
| --- | --- | --- | --- |
|  |  | **Specific aspects of** | **the unidirectional** |
|  | **Common trait** | **the E-SWAN scale** | **scale** |
| **Depression** | 0.338 | 0.089 | 0.156 |
| **Social Anxiety** | 0.455 | 0.058 | 0.254 |
| **Panic** | 0.483 | 0.062 | 0.211 |
| **DMDD** | 0.54 | 0.18 | 0.062 |

7

**Table S3.** Discrimination (α) and difficulty (β) parameters for E-SWAN Depression and Mood and Feelings Questionnaire (MFQ)


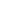

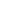

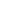

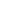

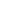

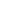

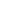

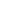

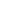

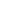

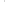


|  | **α General Factor** | **α E-SWAN** | **α MFQ** | **β1** | **β2** | **β3** | **β4** | **β5** | **β6** |
| --- | --- | --- | --- | --- | --- | --- | --- | --- | --- |
| **MDD_1A** | 1.621 | 0.803 | 0 | 5.636 | 3.542 | 2.385 | -1.393 | -2.631 | -4.508 |
| **MDD_1B** | 1.135 | 0.655 | 0 | 4.895 | 3.497 | 2.624 | -0.414 | -1.77 | -3.197 |
| **MDD_2A** | 1.09 | 1.75 | 0 | 4.94 | 2.786 | 1.387 | -1.959 | -3.257 | -4.969 |
| **MDD_2B** | 1.331 | 1.93 | 0 | 4.41 | 2.429 | 0.934 | -2.969 | -4.047 | -6.522 |
| **MDD_3A** | 0.78 | 1.959 | 0 | 4.648 | 2.737 | 1.927 | -2.616 | -3.801 | -4.865 |
| **MDD_3B** | 0.826 | 1.773 | 0 | 4.495 | 2.867 | 1.686 | -1.853 | -3.399 | -5.014 |
| **MDD_4** | 0.596 | 1.483 | 0 | 4.795 | 2.505 | 1.556 | -1.59 | -3.154 | -5.675 |
| **MDD_5** | 0.823 | 1.962 | 0 | 5.458 | 3.266 | 2.249 | -2.672 | -4.508 | -6.437 |
| **MDD_6** | 0.756 | 2.067 | 0 | 4.315 | 2.12 | 1.116 | -2.705 | -4.326 | -6.135 |
| **MDD_7** | 2.083 | 1.236 | 0 | 5.161 | 3.208 | 1.891 | -1.308 | -3.265 | -5.414 |
| **MDD_8A** | 0.981 | 0.598 | 0 | 3.843 | 2.619 | 2.117 | 0.414 | -1.055 | -3.08 |
| **MDD_8B** | 1.255 | 1.118 | 0 | 5.02 | 3.12 | 2.145 | -0.936 | -2.278 | -4.269 |
| **MDD_9** | 2.01 | 1.218 | 0 | 3.508 | 1.822 | 1.055 | -3.333 | -4.765 | -6.488 |
| **MFQ_P_01** | 2.075 | 0 | 1.21 | -0.124 | -3.859 |  |  |  |  |
| **MFQ_P_02** | 1.657 | 0 | 1.688 | -2.498 | -5.778 |  |  |  |  |
| **MFQ_P_03** | 0.773 | 0 | 1.489 | -1.982 | -4.34 |  |  |  |  |
| **MFQ_P_04** | 0.427 | 0 | 1.045 | -1.205 | -3.146 |  |  |  |  |
| **MFQ_P_05** | 0.851 | 0 | 2.211 | -2.153 | -4.353 |  |  |  |  |
| **MFQ_P_06** | 1.146 | 0 | 3.609 | -4.43 | -8.16 |  |  |  |  |
| **MFQ_P_07** | 1.029 | 0 | 1.143 | -1.009 | -3.348 |  |  |  |  |
| **MFQ_P_08** | 2.636 | 0 | 1.243 | -3.035 | -6.752 |  |  |  |  |
| **MFQ_P_09** | 1.65 | 0 | 0.899 | -2.228 | -4.808 |  |  |  |  |
| **MFQ_P_10** | 1.071 | 0 | 1.033 | -0.473 | -2.977 |  |  |  |  |
| **MFQ_P_11** | 1.224 | 0 | 0.966 | 0.7 | -2.4 |  |  |  |  |
| **MFQ_P_12** | 1.023 | 0 | 2.135 | -1.954 | -5.124 |  |  |  |  |
| **MFQ_P_13** | 1.146 | 0 | 4.469 | -7.491 | -10.847 |  |  |  |  |
| **MFQ_P_14** | 1.108 | 0 | 0.872 | -1.57 | -3.619 |  |  |  |  |
| **MFQ_P_15** | 2.611 | 0 | 0.905 | -3.649 | -6.163 |  |  |  |  |
| **MFQ_P_16** | 3.328 | 0 | 0.775 | -5.325 | -8.494 |  |  |  |  |
| **MFQ_P_17** | 2.288 | 0 | 0.361 | -3.837 | -6.276 |  |  |  |  |
| **MFQ_P_18** | 3.491 | 0 | 0.445 | -5.633 | -10.079 |  |  |  |  |
| **MFQ_P_19** | 3.054 | 0 | 0.505 | -5.617 | -9.464 |  |  |  |  |
| **MFQ_P_20** | 1.388 | 0 | 1.254 | -3.426 | -5.93 |  |  |  |  |
| **MFQ_P_21** | 1.094 | 0 | 0.687 | -0.462 | -2.714 |  |  |  |  |
| **MFQ_P_22** | 1.729 | 0 | 0.82 | -2.621 | -5.288 |  |  |  |  |
| **MFQ_P_23** | 3.962 | 0 | 1.066 | -5.26 | -9.208 |  |  |  |  |
| **MFQ_P_24** | 2.79 | 0 | 0.467 | -3.403 | -6.975 |  |  |  |  |
| **MFQ_P_25** | 1.728 | 0 | 0.743 | -2.73 | -5.048 |  |  |  |  |
| **MFQ_P_26** | 0.873 | 0 | 0.757 | -1.923 | -3.823 |  |  |  |  |
| **MFQ_P_27** | 1.492 | 0 | 0.593 | -1.85 | -4.501 |  |  |  |  |
| **MFQ_P_28** | 2.145 | 0 | 0.783 | -2.897 | -5.949 |  |  |  |  |
| **MFQ_P_29** | 1.354 | 0 | 0.991 | -1.009 | -3.521 |  |  |  |  |
| **MFQ_P_30** | 1.938 | 0 | 0.552 | -1.645 | -4.395 |  |  |  |  |
| **MFQ_P_31** | 1.916 | 0 | 1.138 | -2.177 | -6.484 |  |  |  |  |
| **MFQ_P_32** | 0.783 | 0 | 0.99 | -1.473 | -4.285 |  |  |  |  |
| **MFQ_P_33** | 0.55 | 0 | 1.495 | -2.179 | -4.28 |  |  |  |  |
| **MFQ_P_34** | 2.049 | 0 | 2.016 | -3.306 | -6.183 |  |  |  |  |

8

**Table S4.** Discrimination (α) and difficulty (β) parameters for E-SWAN Social Anxiety and Screen for Child Anxiety and Related Disorders (SCARED) Social Anxiety


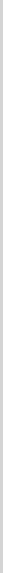

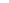

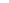

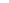

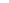

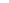

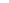

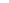

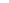

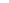

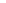


|  | **α General Factor** | **α E-SWAN** | **α SCARED** | **β1** | **β2** | **β3** | **β4** | **β5** | **β6** |
| --- | --- | --- | --- | --- | --- | --- | --- | --- | --- |
| **SocAnx_01** | 1.796 | 2.848 | 0 | 8.174 | 5.212 | 4.263 | -0.967 | -3.092 | -6.071 |
| **SocAnx_02** | 2.231 | 3.594 | 0 | 9.879 | 6.26 | 4.765 | -1.593 | -4.465 | -8.467 |
| **SocAnx_03** | 2.86 | 3.866 | 0 | 11.505 | 6.547 | 4.703 | -1.919 | -5.138 | -8.978 |
| **SocAnx_04A** | 1.132 | 1.324 | 0 | 4.755 | 2.7 | 1.766 | -1.032 | -2.225 | -3.818 |
| **SocAnx_05** | 1.875 | 3.178 | 0 | 9.033 | 5.285 | 3.695 | -1.304 | -3.649 | -6.382 |
| **SCARED_P_03** | 1.72 | 0 | 0.828 | 0.317 | -2.596 |  |  |  |  |
| **SCARED_P_10** | 2.204 | 0 | 0.58 | -0.665 | -4.06 |  |  |  |  |
| **SCARED_P_26** | 3.651 | 0 | 2.173 | -1.104 | -5.171 |  |  |  |  |
| **SCARED_P_32** | 4.665 | 0 | 2.758 | 0.84 | -5.179 |  |  |  |  |
| **SCARED_P_39** | 2.414 | 0 | -0.349 | -0.73 | -2.985 |  |  |  |  |
| **SCARED_P_40** | 3.287 | 0 | -0.689 | -0.809 | -4.47 |  |  |  |  |
| **SCARED_P_41** | 2.299 | 0 | 1.143 | -0.041 | -3.733 |  |  |  |  |

*Due to convergence problems we excluded item 4B from the bifactor analysis

9

**Table S5.** Discrimination (α) and difficulty (β) parameters for E-SWAN Panic Disorder and Screen for Child Anxiety and Related Disorders (SCARED) Panic Disorder


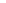

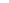

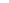

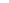

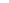

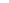

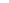

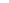

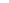

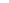

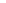


|  | **α General Factor** | **α E-SWAN** | **α SCARED** | **β1** | **β2** | **β3** | **β4** | **β5** | **β6** |
| --- | --- | --- | --- | --- | --- | --- | --- | --- | --- |
| **Panic_B01** | 3.467 | 1.719 | 0 | 7.403 | 4.858 | 3.542 | -3.776 | -5.908 | -8.409 |
| **Panic_B02** | 3.82 | 2.083 | 0 | 8.053 | 5.274 | 3.327 | -5.25 | -7.379 | -8.646 |
| **Panic_B03** | 3.526 | 1.653 | 0 | 7.387 | 4.59 | 2.999 | -3.95 | -5.719 | -7.717 |
| **Panic_B04** | 5.086 | 2.203 | 0 | 9.869 | 6.383 | 4.022 | -5.414 | -8.211 | -11.585 |
| **Panic_B05** | 7.895 | -0.385 | 0 | 10.891 | 7.692 | 4.907 | -11.085 | -13.75 | -16.814 |
| **Panic_B06** | 13.083 | -1.014 | 0 | 17.507 | 12.211 | 8.118 | -16.907 | -21.509 | -26.494 |
| **Panic_B07** | 3.386 | 0.509 | 0 | 6.249 | 4.255 | 2.8 | -3.148 | -5.134 | -7.324 |
| **Panic_B08** | 3.642 | 2.271 | 0 | 8.242 | 5.771 | 4.091 | -2.558 | -4.803 | -7.941 |
| **Panic_B09** | 4.122 | 1.805 | 0 | 8.096 | 5.412 | 3.462 | -5.176 | -7.792 | -9.643 |
| **Panic_B10** | 4.705 | 0.651 | 0 | 7.7 | 4.862 | 3.453 | -7.632 | -11.109 |  |
| **Panic_B11** | 3.464 | 1.624 | 0 | 6.789 | 4.6 | 3.018 | -3.93 | -5.98 | -8.301 |
| **Panic_B12** | 3.815 | 2.377 | 0 | 7.961 | 5.533 | 3.736 | -2.75 | -5.336 | -8.456 |
| **Panic_B13** | 3.637 | 1.506 | 0 | 6.415 | 4.19 | 2.69 | -3.689 | -5.639 | -7.948 |
| **SCARED_P_01** | 1.49 | 0 | 2.398 | -3.321 | -5.929 |  |  |  |  |
| **SCARED_P_06** | 2.984 | 0 | 3.298 | -8.549 | -10.948 |  |  |  |  |
| **SCARED_P_09** | 1.142 | 0 | 0.806 | -3.048 | -6.204 |  |  |  |  |
| **SCARED_P_12** | 1.124 | 0 | 1.843 | -3.537 | -5.367 |  |  |  |  |
| **SCARED_P_15** | 0.988 | 0 | 1.176 | -3.604 | -5.632 |  |  |  |  |
| **SCARED_P_18** | 0.815 | 0 | 2.059 | -0.424 | -3.666 |  |  |  |  |
| **SCARED_P_19** | 1.014 | 0 | 2.26 | -2.971 | -5.835 |  |  |  |  |
| **SCARED_P_22** | 0.649 | 0 | 1.722 | -3.323 | -5.207 |  |  |  |  |
| **SCARED_P_24** | 0.888 | 0 | 1.08 | -2.355 | -4.186 |  |  |  |  |
| **SCARED_P_27** | 1.81 | 0 | 2.848 | -6.514 | -8.928 |  |  |  |  |
| **SCARED_P_30** | 1.987 | 0 | 2.472 | -4.376 | -6.863 |  |  |  |  |
| **SCARED_P_34** | 1.187 | 0 | 1.85 | -3.747 | -5.483 |  |  |  |  |
| **SCARED_P_38** | 2.186 | 0 | 1.905 | -5.061 | -7.385 |  |  |  |  |

10

**Table S6.** Discrimination (α) and difficulty (β) parameters for E-SWAN DMDD and Affective Reactivity Index (ARI)


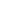

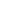

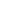

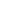

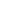

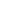

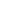

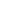

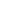

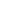

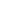


|  | **α General Factor** | **α E-SWAN** | **α ARI** | **β1** | **β2** | **β3** | **β4** | **β5** | **β6** |
| --- | --- | --- | --- | --- | --- | --- | --- | --- | --- |
| **DMDD_1A** | 3.41 | -0.09 | 0 | 7.297 | 4.721 | 3.639 | 0.226 | -2.109 | -4.85 |
| **DMDD_1B** | 2.506 | 1.932 | 0 | 5.26 | 2.953 | 1.713 | -2.113 | -4.151 | -6.93 |
| **DMDD_1C** | 2.539 | 2.894 | 0 | 5.32 | 2.837 | 1.466 | -2.708 | -4.513 | -7.271 |
| **DMDD_2A** | 2.879 | 0.014 | 0 | 5.08 | 3.427 | 2.357 | -1.024 | -2.712 | -5.056 |
| **DMDD_2B** | 2.617 | 2.37 | 0 | 4.648 | 2.354 | 0.928 | -3.559 | -5.385 | -8.301 |
| **DMDD_2C** | 2.376 | 2.804 | 0 | 4.375 | 2.143 | 0.622 | -3.655 | -5.577 | -7.695 |
| **DMDD_3A** | 2.175 | 0.393 | 0 | 3.274 | 1.846 | 1.228 | -1.297 | -2.788 | -4.331 |
| **DMDD_3B** | 2.061 | 1.962 | 0 | 3.327 | 1.365 | 0.485 | -3.511 | -4.96 | -6.428 |
| **DMDD_3C** | 2.08 | 2.439 | 0 | 3.618 | 1.293 | 0.303 | -3.813 | -5.155 | -6.722 |
| **DMDD_4A** | 4.288 | -0.228 | 0 | 7.376 | 4.957 | 3.621 | -0.416 | -3.104 | -6.086 |
| **DMDD_4B** | 3.063 | 2.418 | 0 | 5.208 | 2.912 | 1.409 | -3.225 | -5.571 | -8.447 |
| **DMDD_4C** | 3.194 | 3.481 | 0 | 5.968 | 3.171 | 1.392 | -3.476 | -5.803 | -8.751 |
| **DMDD_5A** | 2.351 | 0.016 | 0 | 5.066 | 3.37 | 2.298 | -0.643 | -2.362 | -4.081 |
| **DMDD_5B** | 1.969 | 1.22 | 0 | 4.39 | 2.794 | 1.797 | -1.765 | -3.413 | -5.078 |
| **DMDD_5C** | 1.848 | 1.643 | 0 | 4.77 | 2.816 | 1.876 | -1.575 | -3.11 | -5.108 |
| **DMDD_6A** | 4.137 | -0.288 | 0 | 8.801 | 6.311 | 4.629 | 0.198 | -2.141 | -5.701 |
| **DMDD_6B** | 2.807 | 1.81 | 0 | 6.459 | 4.148 | 2.671 | -1.814 | -4.265 | -7.293 |
| **DMDD_6C** | 2.772 | 2.721 | 0 | 7.263 | 4.476 | 2.616 | -2.133 | -4.406 | -7.31 |
| **DMDD_7A** | 4.694 | -0.397 | 0 | 9.913 | 6.728 | 5.199 | -0.095 | -3.151 | -6.994 |
| **DMDD_7B** | 2.798 | 1.764 | 0 | 6.635 | 3.988 | 2.761 | -2.069 | -4.237 | -7.78 |
| **DMDD_7C** | 2.29 | 2.144 | 0 | 6.042 | 3.632 | 2.407 | -1.512 | -3.599 | -6.1 |
| **DMDD_8A** | 4.024 | -0.252 | 0 | 7.218 | 4.68 | 3.469 | -0.459 | -2.699 | -5.732 |
| **DMDD_8B** | 3.238 | 2.319 | 0 | 5.81 | 3.32 | 1.82 | -3.561 | -6.068 | -8.44 |
| **DMDD_8C** | 3.17 | 3.222 | 0 | 6.298 | 3.431 | 1.775 | -3.235 | -5.853 | -8.38 |
| **DMDD_9A** | 2.364 | -0.046 | 0 | 4.525 | 2.706 | 1.654 | -1.737 | -3.319 | -4.904 |
| **DMDD_9B** | 2.098 | 1.128 | 0 | 4.257 | 2.232 | 1.145 | -3.127 | -4.693 | -6.388 |
| **DMDD_9C** | 1.908 | 1.539 | 0 | 4.609 | 2.346 | 1.138 | -2.31 | -3.807 | -5.931 |
| **DMDD_10A** | 3.638 | -0.254 | 0 | 6.573 | 4.458 | 3.19 | -0.807 | -3.153 | -5.789 |
| **DMDD_10B** | 2.614 | 1.487 | 0 | 4.75 | 3.1 | 1.88 | -2.413 | -4.637 | -6.545 |
| **DMDD_10C** | 2.208 | 2.078 | 0 | 4.908 | 2.999 | 1.686 | -1.75 | -3.78 | -5.726 |
| **ARI_P_01** | 1.635 | 0 | 1.905 | 0.724 | -2.989 |  |  |  |  |
| **ARI_P_02** | 4.182 | 0 | 3.272 | 0.762 | -5.214 |  |  |  |  |
| **ARI_P_03** | 1.937 | 0 | 1.69 | -1.382 | -4.277 |  |  |  |  |
| **ARI_P_04** | 1.769 | 0 | 1.971 | -3.313 | -6.582 |  |  |  |  |
| **ARI_P_05** | 4.31 | 0 | 3.26 | -1.645 | -7.624 |  |  |  |  |
| **ARI_P_06** | 3.407 | 0 | 2.34 | -0.105 | -4.719 |  |  |  |  |
| **ARI_P_07** | 2.857 | 0 | 1.995 | -0.504 | -4.048 |  |  |  |  |

11

**Figure S5.** Test Information Function Plots.

Plots depict the overall performance of each E-SWAN scale and its unidirectional counterpart (Mood and Feelings Questionnaire [MFQ], Screen for Child Anxiety and Related Disorders [SCARED], Affective Reactivity Index [ARI]) at measuring the latent trait for each scale modeled independently. Brackets represent areas of the curve where information is reliable. See supplementary tables 2-9 for specific values for each measure.


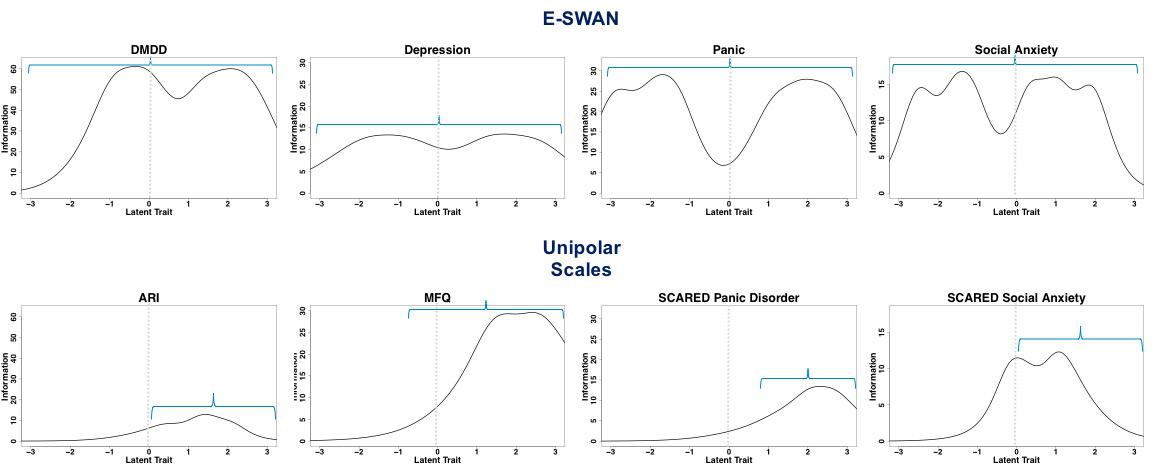


12

**Figure S6.** Item Information Curves*.*

Plots depict the performance of each item on each of the E-SWAN scales and its unidirectional counterpart at measuring the latent trait (Mood and Feelings Questionnaire [MFQ], Screen for Child Anxiety and Related Disorders [SCARED], Affective Reactivity Index [ARI]).


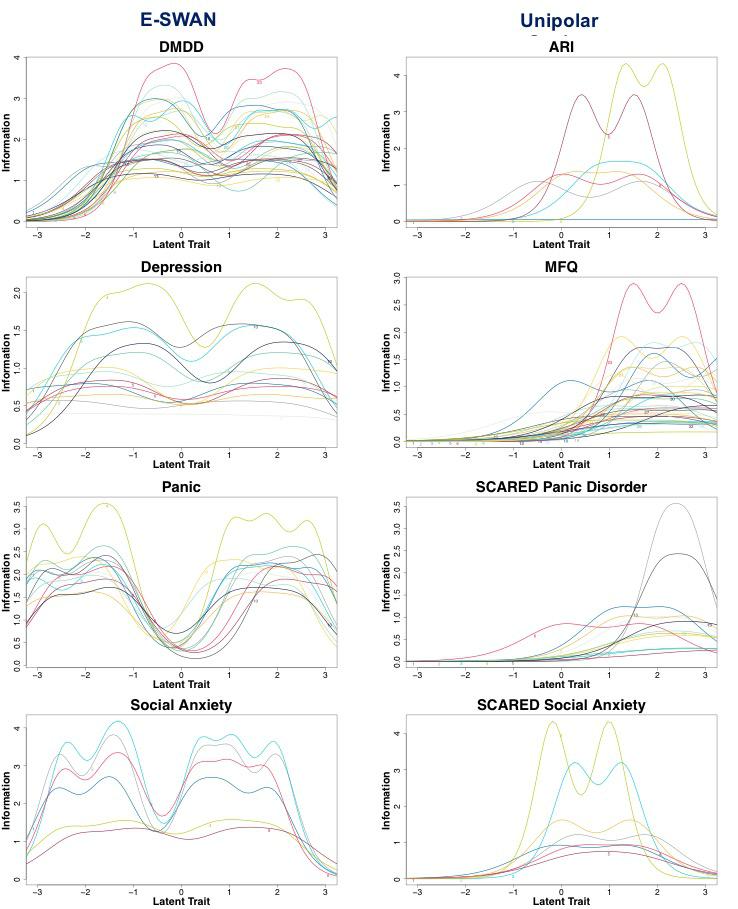


13

**Figure S7.** Sample Item Characteristic Curves.

An example Item Characteristic Curve from the E-SWAN Depression scale, and the Mood and Feelings Questionnaire (MFQ) are shown. Lines represent the probability of endorsing a specific response choice at each area of the latent trait.


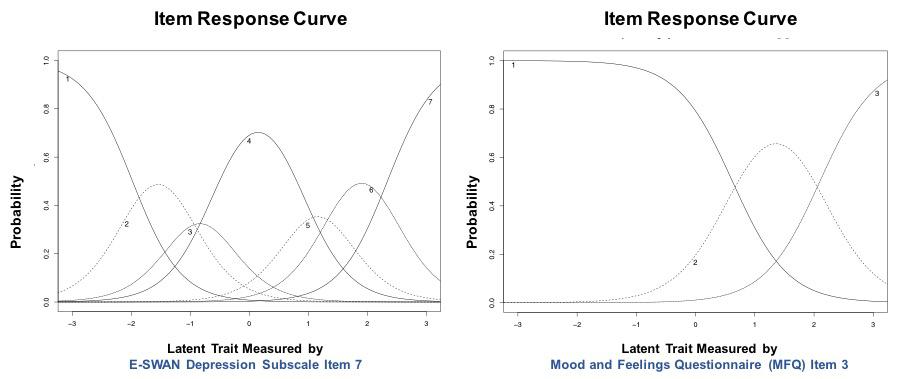


14

**Figure S8.** ROC Curves for All Scales.

ROC Curves are depicted for each diagnosis as predicted by each E-SWAN and unidirectional scale, showing specificity for each of the E-SWAN scales and their respective DSM-5 diagnoses. (Mood and Feelings Questionnaire [MFQ], Screen for Child Anxiety and Related Disorders [SCARED], Affective Reactivity Index [ARI]).


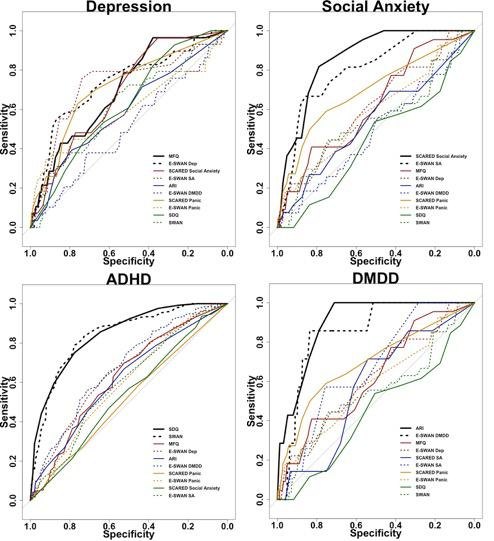


15

**Figure S9.** Distribution of E-SWAN Scores by Diagnosis.


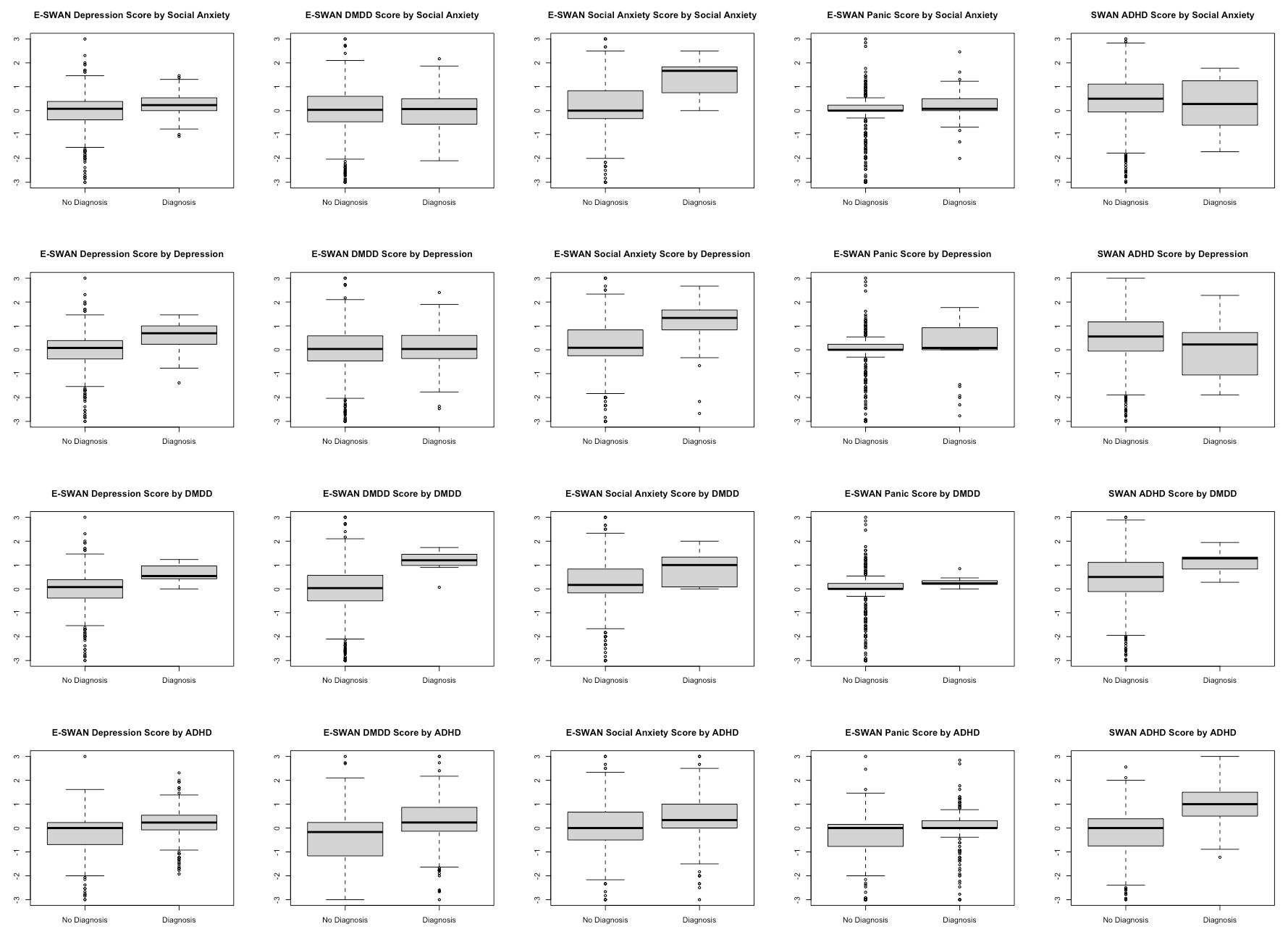


16

**Figure S10.** Distribution of SDQ Prosocial scores by E-SWAN score quintile.

Each E-SWAN score divided into quintiles and plotted against

the prosocial behaviors subscale of the Strengths and Difficulties Questionnaire (SDQ).


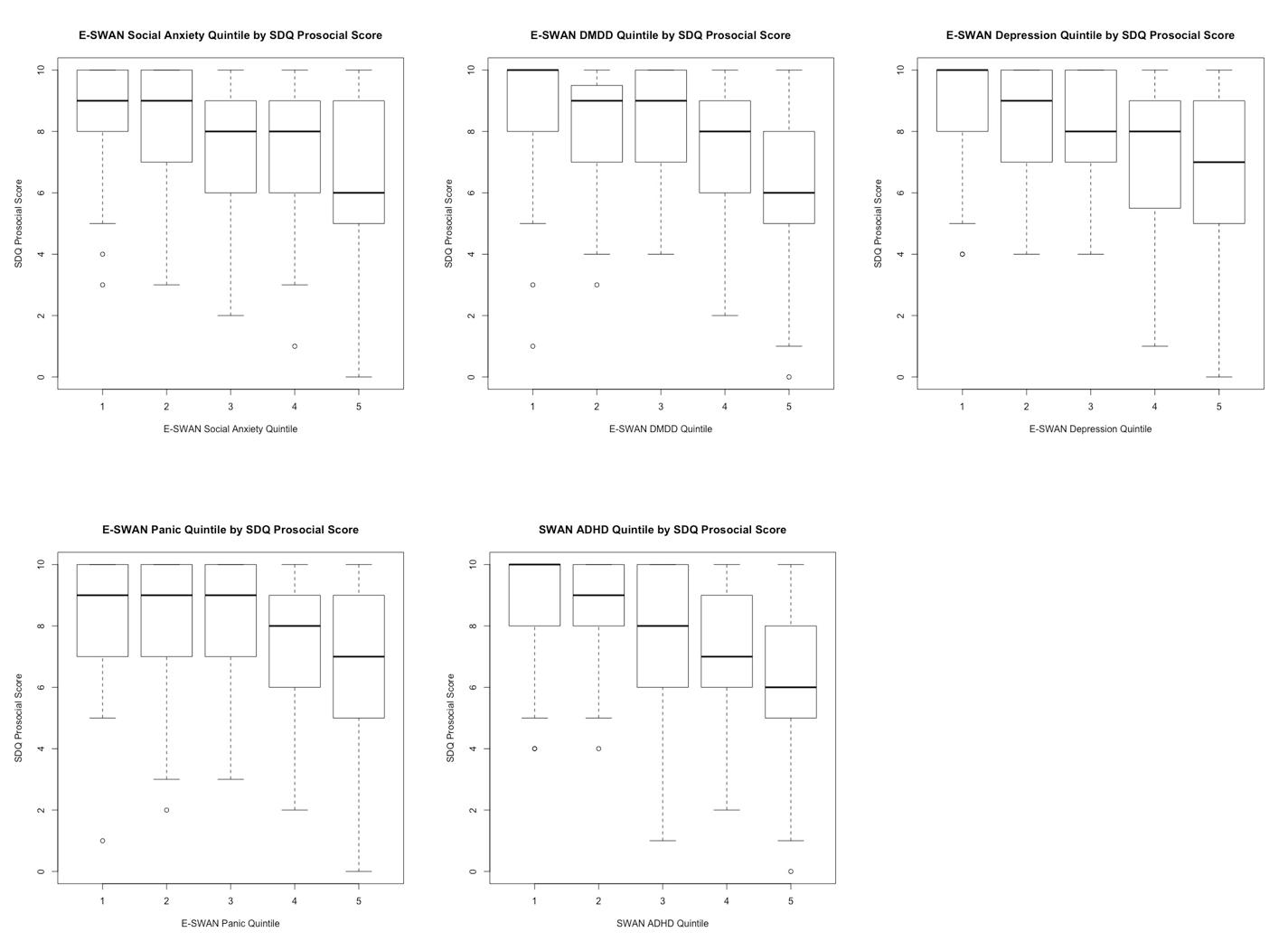


17

**Table S7.** Measures of Fit for Confirmatory Factor Analysis.

|  | **Comparative** | **Tucker-Lewis** |  |
| --- | --- | --- | --- |
|  | **Fit Index (CFI)** | **Index (TLI)** | **RMSEA** |
| **E-SWAN Depression** | 0.99** | 0.99** | 0.085 |
| **E-SWAN Social Anxiety** | 1.00** | 1.00** | 0.014** |
| **E-SWAN Panic Disorder** | 0.99** | 0.99** | 0.082 |
| **E-SWAN DMDD** | 0.99** | 0.99** | 0.059** |
| **ARI** | 1.00** | 0.99** | 0.035** |
| **MFQ** | 0.98** | 0.98** | 0.056** |
| **SCARED Panic** | 0.99** | 0.99** | 0.022** |
| **SCARED Social** | 0.99** | 0.99** | 0.037** |
| ****good; *acceptable** |  |  |  |

**Table S8.** Monotonicity results*.*

We tested the assumption of monotonicity within each of the scales utilizing the check. monotonicity function in the mokken R package (van der Ark, 2012). This method involves dividing the sample into rest score groups and then examining the item step response functions for instances in which they fail to be nondecreasing with the latent trait. A rest score for an item is the score on all items except that item. This test gives the number of active pairs (#ac); the number of violations of manifest monotonicity (#vi); the average number of violations of manifest monotonicity per active pair (#vi/#ac), the largest violation of manifest monotonicity (maxvi); the sum of violations of manifest monotonicity (sum); the average violation per active pair (sum/#ac); the two rest score groups that are involved in the largest violation of manifest monotonicity; and the number of violations that are significantly greater than zero. A majority of the items, with a few exceptions, met the assumptions of monotonicity.


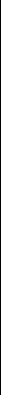

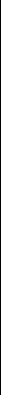


**Monotonicity of E-SWAN Social Anxiety Items**

|  | **ItemH** | **#ac** | **#vi** | **#vi/#ac** | **maxvi** | **sum** | **sum/#ac** | **zmax** | **#zsig** | **crit** |
| --- | --- | --- | --- | --- | --- | --- | --- | --- | --- | --- |
| SocAnx_01 | 0.68 | 88 | 3 | 0.03 | 0.04 | 0.11 | 0.0012 | 0.84 | 0 | -2 |
| SocAnx_02 | 0.71 | 60 | 3 | 0.05 | 0.05 | 0.12 | 0.002 | 1.19 | 0 | 2 |
| SocAnx_03 | 0.75 | 58 | 0 | 0 | 0 | 0 | 0 | 0 | 0 | 0 |
| SocAnx_04A | 0.68 | 75 | 1 | 0.01 | 0.04 | 0.04 | 0.0005 | 0.66 | 0 | -8 |
| SocAnx_04B | 0.69 | 73 | 2 | 0.03 | 0.08 | 0.13 | 0.0018 | 0.99 | 0 | 3 |
| SocAnx_05 | 0.73 | 76 | 0 | 0 | 0 | 0 | 0 | 0 | 0 | 0 |
|  |  |  | |  |  |  |  |  |  |  |
|  |  | **Monotonicity of E-SWAN Panic Items** | | | | | |  |  |  |
|  | **ItemH** | **#ac** | **#vi** | **#vi/#ac** | **maxvi** | **sum** | **sum/#ac** | **zmax** | **#zsig** | **crit** |

18

| Panic_B01 | 0.78 | 36 | 0 | 0 | 0 | 0 | 0 | 0 | 0 | 0 |
| --- | --- | --- | --- | --- | --- | --- | --- | --- | --- | --- |
| Panic_B02 | 0.79 | 37 | 1 | 0.03 | 0.03 | 0.03 | 0.0009 | 0.88 | 0 | -11 |
| Panic_B03 | 0.78 | 40 | 2 | 0.05 | 0.05 | 0.09 | 0.0024 | 1.2 | 0 | -2 |
| Panic_B04 | 0.83 | 21 | 0 | 0 | 0 | 0 | 0 | 0 | 0 | 0 |
| Panic_B05 | 0.8 | 38 | 0 | 0 | 0 | 0 | 0 | 0 | 0 | 0 |
| Panic_B06 | 0.81 | 41 | 0 | 0 | 0 | 0 | 0 | 0 | 0 | 0 |
| Panic_B07 | 0.75 | 61 | 0 | 0 | 0 | 0 | 0 | 0 | 0 | 0 |
| Panic_B08 | 0.79 | 41 | 1 | 0.02 | 0.03 | 0.03 | 0.0008 | 0.67 | 0 | -12 |
| Panic_B09 | 0.81 | 28 | 0 | 0 | 0 | 0 | 0 | 0 | 0 | 0 |
| Panic_B10 | 0.82 | 50 | 0 | 0 | 0 | 0 | 0 | 0 | 0 | 0 |
| Panic_B11 | 0.78 | 34 | 1 | 0.03 | 0.04 | 0.04 | 0.0012 | 0.86 | 0 | -9 |
| Panic_B12 | 0.8 | 36 | 2 | 0.06 | 0.05 | 0.09 | 0.0024 | 1.88 | 1 | 14 |
| Panic_B13 | 0.78 | 41 | 0 | 0 | 0 | 0 | 0 | 0 | 0 | 0 |
|  |  |  | | | | | |  |  |  |
|  |  | **Monotonicity of E-SWAN Depression Items** | | | | | |  |  |  |
|  | **ItemH** | **#ac** | **#vi** | **#vi/#ac** | **maxvi** | **sum** | **sum/#ac** | **zmax** | **#zsig** | **crit** |
| MDD_1A | 0.42 | 134 | 6 | 0.04 | 0.05 | 0.25 | 0.0018 | 0.81 | 0 | 17 |
| MDD_1B | 0.4 | 141 | 4 | 0.03 | 0.06 | 0.18 | 0.0013 | 0.8 | 0 | 15 |
| MDD_2A | 0.48 | 131 | 1 | 0.01 | 0.04 | 0.04 | 0.0003 | 0.34 | 0 | 0 |
| MDD_2B | 0.53 | 92 | 0 | 0 | 0 | 0 | 0 | 0 | 0 | 0 |
| MDD_3A | 0.45 | 122 | 13 | 0.11 | 0.08 | 0.57 | 0.0047 | 1.93 | 1 | 47 |
| MDD_3B | 0.44 | 141 | 9 | 0.06 | 0.11 | 0.49 | 0.0034 | 1.35 | 0 | 30 |
| MDD_4 | 0.41 | 116 | 5 | 0.04 | 0.06 | 0.21 | 0.0018 | 1.03 | 0 | 19 |
| MDD_5 | 0.48 | 114 | 3 | 0.03 | 0.05 | 0.11 | 0.001 | 0.46 | 0 | 6 |
| MDD_6 | 0.47 | 120 | 9 | 0.08 | 0.12 | 0.64 | 0.0054 | 1.83 | 1 | 47 |
| MDD_7 | 0.51 | 110 | 3 | 0.03 | 0.1 | 0.2 | 0.0018 | 1.96 | 1 | 30 |
| MDD_8A | 0.38 | 113 | 9 | 0.08 | 0.14 | 0.66 | 0.0059 | 1.82 | 1 | 54 |
| MDD_8B | 0.48 | 123 | 3 | 0.02 | 0.08 | 0.15 | 0.0012 | 0.96 | 0 | 13 |
| MDD_9 | 0.49 | 100 | 3 | 0.03 | 0.06 | 0.17 | 0.0017 | 1.12 | 0 | 12 |


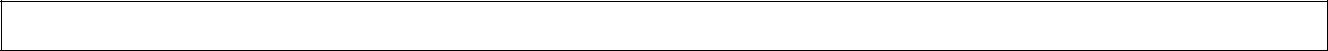


**Monotonicity of E-SWAN DMDD Items**

19

|  | **ItemH** | **#ac** | **#vi** | **#vi/#ac** | **maxvi** | **sum** | **sum/#ac** | **zmax** | **#zsig** | **crit** |
| --- | --- | --- | --- | --- | --- | --- | --- | --- | --- | --- |
| DMDD_1A | 0.63 | 134 | 6 | 0.04 | 0.16 | 0.49 | 0.0037 | 1.56 | 0 | 24 |
| DMDD_1B | 0.66 | 99 | 4 | 0.04 | 0.14 | 0.35 | 0.0036 | 1.97 | 2 | 37 |
| DMDD_1C | 0.65 | 144 | 3 | 0.02 | 0.11 | 0.23 | 0.0016 | 1.51 | 0 | 11 |
| DMDD_2A | 0.62 | 163 | 7 | 0.04 | 0.19 | 0.61 | 0.0038 | 2.25 | 1 | 43 |
| DMDD_2B | 0.69 | 99 | 6 | 0.06 | 0.14 | 0.47 | 0.0048 | 1.75 | 1 | 34 |
| DMDD_2C | 0.66 | 122 | 10 | 0.08 | 0.14 | 0.73 | 0.006 | 1.71 | 1 | 41 |
| DMDD_3A | 0.58 | 185 | 7 | 0.04 | 0.07 | 0.34 | 0.0018 | 0.76 | 0 | 11 |
| DMDD_3B | 0.65 | 116 | 2 | 0.02 | 0.05 | 0.09 | 0.0008 | 0.73 | 0 | -3 |
| DMDD_3C | 0.65 | 141 | 8 | 0.06 | 0.05 | 0.34 | 0.0024 | 0.9 | 0 | 9 |
| DMDD_4A | 0.65 | 128 | 3 | 0.02 | 0.06 | 0.15 | 0.0012 | 0.5 | 0 | -1 |
| DMDD_4B | 0.7 | 88 | 3 | 0.03 | 0.04 | 0.12 | 0.0013 | 0.67 | 0 | -3 |
| DMDD_4C | 0.69 | 135 | 1 | 0.01 | 0.06 | 0.06 | 0.0004 | 0.86 | 0 | -5 |
| DMDD_5A | 0.58 | 170 | 5 | 0.03 | 0.11 | 0.37 | 0.0022 | 1.22 | 0 | 16 |
| DMDD_5B | 0.63 | 125 | 4 | 0.03 | 0.05 | 0.17 | 0.0014 | 0.57 | 0 | 2 |
| DMDD_5C | 0.62 | 139 | 4 | 0.03 | 0.06 | 0.16 | 0.0012 | 0.86 | 0 | 3 |
| DMDD_6A | 0.64 | 118 | 8 | 0.07 | 0.24 | 0.73 | 0.0062 | 2.47 | 2 | 59 |
| DMDD_6B | 0.69 | 91 | 3 | 0.03 | 0.09 | 0.19 | 0.002 | 1.06 | 0 | 6 |
| DMDD_6C | 0.68 | 103 | 4 | 0.04 | 0.08 | 0.19 | 0.0018 | 1.29 | 0 | 7 |
| DMDD_7A | 0.65 | 126 | 5 | 0.04 | 0.15 | 0.4 | 0.0032 | 1.52 | 0 | 20 |
| DMDD_7B | 0.7 | 79 | 3 | 0.04 | 0.13 | 0.21 | 0.0027 | 1.65 | 1 | 25 |
| DMDD_7C | 0.65 | 134 | 4 | 0.03 | 0.07 | 0.2 | 0.0015 | 0.74 | 0 | 4 |
| DMDD_8A | 0.63 | 166 | 6 | 0.04 | 0.24 | 0.59 | 0.0036 | 2.5 | 1 | 47 |
| DMDD_8B | 0.7 | 121 | 0 | 0 | 0 | 0 | 0 | 0 | 0 | 0 |
| DMDD_8C | 0.68 | 112 | 6 | 0.05 | 0.05 | 0.25 | 0.0022 | 0.77 | 0 | 5 |
| DMDD_9A | 0.57 | 174 | 8 | 0.05 | 0.09 | 0.43 | 0.0025 | 0.97 | 0 | 16 |
| DMDD_9B | 0.65 | 110 | 2 | 0.02 | 0.09 | 0.15 | 0.0014 | 1.78 | 1 | 19 |
| DMDD_9C | 0.62 | 155 | 2 | 0.01 | 0.05 | 0.09 | 0.0006 | 0.64 | 0 | -2 |
| DMDD_10A | 0.63 | 133 | 8 | 0.06 | 0.17 | 0.63 | 0.0048 | 2.15 | 2 | 48 |
| DMDD_10B | 0.68 | 84 | 3 | 0.04 | 0.1 | 0.23 | 0.0027 | 1.59 | 0 | 11 |
| DMDD_10C | 0.64 | 133 | 7 | 0.05 | 0.1 | 0.4 | 0.003 | 1.5 | 0 | 17 |

20

**Table S9.** Item Response Theory Parameters for E-SWAN Depression Scale.

|  |  | Discrimination (α) and difficulty (β) parameters | | | | | |  |  | Item information by area of the latent trait (z-score) | | | | | | |
| --- | --- | --- | --- | --- | --- | --- | --- | --- | --- | --- | --- | --- | --- | --- | --- | --- |
|  | α | β1 | β2 | β3 | β4 | β5 | β6 | Mean(β) | -3 | | -2 | -1 | 0 | 1 | 2 | 3 |
| MDD_1A | 1.612 | -3.237 | -2.036 | -1.364 | 0.872 | 1.534 | 2.614 | -0.270 | 0.37 | | 0.48 | 0.54 | 0.55 | 0.52 | 0.44 | 0.32 |
| MDD_1B | 1.35 | -3.488 | -2.546 | -1.771 | 0.344 | 1.33 | 2.45 | -0.614 | 0.34 | | 0.42 | 0.47 | 0.47 | 0.42 | 0.35 | 0.25 |
| MDD_2A | 2.251 | -2.185 | -1.207 | -0.579 | 0.984 | 1.564 | 2.282 | 0.143 | 0.51 | | 0.77 | 0.86 | 0.83 | 0.86 | 0.83 | 0.62 |
| MDD_2B | 2.654 | -1.705 | -0.884 | -0.283 | 1.254 | 1.71 | 2.666 | 0.460 | 0.48 | | 0.83 | 0.97 | 0.86 | 0.83 | 0.95 | 0.87 |
| MDD_3A | 1.661 | -2.381 | -1.346 | -0.963 | 1.408 | 2.066 | 2.742 | 0.254 | 0.33 | | 0.46 | 0.55 | 0.58 | 0.57 | 0.51 | 0.4 |
| MDD_3B | 1.566 | -2.431 | -1.462 | -0.93 | 1.075 | 2.052 | 2.971 | 0.213 | 0.34 | | 0.47 | 0.56 | 0.59 | 0.57 | 0.5 | 0.38 |
| MDD_4 | 1.467 | -2.91 | -1.541 | -1.006 | 1.138 | 2.132 | 3.744 | 0.260 | 0.3 | | 0.39 | 0.45 | 0.47 | 0.46 | 0.4 | 0.32 |
| MDD_5 | 1.822 | -2.631 | -1.581 | -1.106 | 1.353 | 2.234 | 3.303 | 0.262 | 0.36 | | 0.48 | 0.54 | 0.54 | 0.53 | 0.5 | 0.41 |
| MDD_6 | 1.988 | -1.908 | -0.979 | -0.5 | 1.333 | 2.105 | 2.971 | 0.504 | 0.35 | | 0.56 | 0.69 | 0.72 | 0.71 | 0.68 | 0.56 |
| MDD_7 | 2.293 | -2.064 | -1.24 | -0.712 | 0.626 | 1.37 | 2.185 | 0.028 | 0.54 | | 0.82 | 0.92 | 0.88 | 0.86 | 0.77 | 0.56 |
| MDD_8A | 1.125 | -3.354 | -2.37 | -1.788 | -0.378 | 0.96 | 2.779 | -0.692 | 0.3 | | 0.37 | 0.41 | 0.41 | 0.36 | 0.3 | 0.22 |
| MDD_8B | 1.766 | -2.817 | -1.719 | -1.164 | 0.577 | 1.373 | 2.487 | -0.211 | 0.46 | | 0.62 | 0.7 | 0.7 | 0.66 | 0.55 | 0.39 |
| MDD_9 | 2.083 | -1.474 | -0.681 | -0.369 | 1.558 | 2.149 | 2.862 | 0.674 | 0.32 | | 0.56 | 0.75 | 0.79 | 0.77 | 0.77 | 0.66 |
| Mean | 1.818 | -2.507 | -1.507 | -0.964 | 0.934 | 1.737 | 2.774 | 0.078 |  |  |  |  |  |  |  |  |
| Test information |  |  |  |  |  |  |  |  | 4.99 | | 7.23 | 8.4 | 8.39 | 8.12 | 7.55 | 5.98 |
| SEM |  |  |  |  |  |  |  |  | 0.45 | | 0.37 | 0.34 | 0.35 | 0.35 | 0.36 | 0.41 |
| Reliability |  |  |  |  |  |  |  |  | 0.8 | | 0.86 | 0.88 | 0.88 | 0.88 | 0.87 | 0.83 |

*Model adjusted for residual correlations between 1) 1A and 1B; 2) 2A and 2B; 3) 3A and 3B; 4) 8A and 8B

**Table S10.** Item Response Theory Parameters for E-SWAN Panic Disorder Scale.

|  |  | Discrimination (α) and difficulty (β) parameters | | | | | |  |  | Item information by area of the latent trait (z-score) | | | | | | |
| --- | --- | --- | --- | --- | --- | --- | --- | --- | --- | --- | --- | --- | --- | --- | --- | --- |
|  | α | β1 | β2 | β3 | β4 | β5 | β6 | Mean(β) | -3 | | -2 | -1 | 0 | 1 | 2 | 3 |
| Panic_B01 | 2.818 | -3.060 | -2.028 | -1.504 | 1.066 | 1.892 | 2.793 | -0.140 | 1.14 | | 1.53 | 0.95 | 0.42 | 0.52 | 0.93 | 1.14 |
| Panic_B02 | 2.812 | -2.982 | -1.945 | -1.288 | 1.325 | 2.087 | 2.557 | -0.041 | 0.77 | | 1.3 | 1.14 | 0.71 | 0.78 | 1.05 | 1.04 |
| Panic_B03 | 2.729 | -3.105 | -1.863 | -1.390 | 1.018 | 1.769 | 2.511 | -0.177 | 1.01 | | 1.33 | 0.95 | 0.72 | 0.91 | 1.01 | 0.87 |
| Panic_B04 | 3.488 | -2.912 | -1.840 | -1.323 | 1.037 | 1.806 | 2.683 | -0.091 | 0.84 | | 1.28 | 0.99 | 0.51 | 0.47 | 0.82 | 1.11 |
| Panic_B05 | 2.502 | -2.571 | -1.725 | -1.211 | 1.752 | 2.316 | 3.137 | 0.283 | 0.92 | | 1.35 | 1.04 | 0.78 | 0.92 | 1 | 0.87 |
| Panic_B06 | 2.692 | -2.574 | -1.680 | -1.172 | 1.599 | 2.155 | 3.048 | 0.229 | 0.86 | | 1.3 | 1 | 0.58 | 0.68 | 1.02 | 1.07 |
| Panic_B07 | 2.315 | -3.013 | -2.063 | -1.476 | 1.034 | 1.746 | 2.553 | -0.203 | 0.89 | | 1.24 | 0.92 | 0.53 | 0.62 | 0.9 | 1 |
| Panic_B08 | 2.835 | -3.076 | -2.256 | -1.645 | 0.575 | 1.291 | 2.222 | -0.482 | 0.83 | | 1.2 | 0.95 | 0.61 | 0.71 | 0.99 | 1.01 |

21

| Panic_B09 | 2.974 | -2.906 | -1.855 | -1.327 | 1.337 | 2.139 | 2.748 | 0.023 | 0.81 | 1.16 | 0.92 | 0.53 | 0.54 | 0.88 | 1.08 |
| --- | --- | --- | --- | --- | --- | --- | --- | --- | --- | --- | --- | --- | --- | --- | --- |
| Panic_B10 | 2.847 | -2.784 | -1.742 | -1.337 | 1.913 | 2.735 | 3.090 | 0.313 | 0.62 | 1.09 | 1.05 | 0.62 | 0.5 | 0.79 | 1.06 |
| Panic_B11 | 2.603 | -2.858 | -1.944 | -1.382 | 1.078 | 1.703 | 2.490 | -0.152 | 0.75 | 1.12 | 0.96 | 0.67 | 0.74 | 0.94 | 0.9 |
| Panic_B12 | 2.564 | -2.896 | -2.035 | -1.447 | 0.549 | 1.320 | 2.320 | -0.365 | 0.59 | 1.02 | 0.99 | 0.61 | 0.49 | 0.75 | 1 |
| Panic_B13 | 2.390 | -2.671 | -1.649 | -1.184 | 0.958 | 1.683 | 2.469 | -0.066 | 0.61 | 1 | 0.93 | 0.55 | 0.41 | 0.62 | 0.95 |
| Mean | 2.736 | -2.878 | -1.894 | -1.360 | 1.172 | 1.896 | 2.663 | -0.067 |  |  |  |  |  |  |  |
| Test information |  |  |  |  |  |  |  |  | 10.66 | 15.93 | 12.79 | 7.84 | 8.29 | 11.69 | 13.08 |
| SEM |  |  |  |  |  |  |  |  | 0.31 | 0.25 | 0.28 | 0.36 | 0.35 | 0.29 | 0.28 |
| Reliability |  |  |  |  |  |  |  |  | 0.91 | 0.94 | 0.92 | 0.87 | 0.88 | 0.91 | 0.92 |

*Model adjusted for residual correlations between items 5, 6, and 10; items 12 and 13; items 2 and 3

**Table S11.** Item Response Theory Parameters for E-SWAN Social Anxiety Scale.

|  |  | Discrimination (α) and difficulty (β) parameters | | | | | |  |  | Item information by area of the latent trait (z-score) | | | | | | |
| --- | --- | --- | --- | --- | --- | --- | --- | --- | --- | --- | --- | --- | --- | --- | --- | --- |
|  | α | β1 | β2 | β3 | β4 | β5 | β6 | Mean(β) | -3 | | -2 | -1 | 0 | 1 | 2 | 3 |
| SocAnx_01 | 3.035 | -2.621 | -1.618 | -1.340 | 0.266 | 0.888 | 1.863 | -0.427 | 1.4 | | 1.45 | 0.76 | 0.76 | 1.12 | 1.15 | 0.99 |
| SocAnx_02 | 3.584 | -2.569 | -1.578 | -1.239 | 0.377 | 1.036 | 1.998 | -0.329 | 1.3 | | 1.41 | 0.92 | 0.92 | 1.17 | 1.13 | 0.93 |
| SocAnx_03 | 3.746 | -2.412 | -1.512 | -1.108 | 0.370 | 1.086 | 1.958 | -0.270 | 1.15 | | 1.26 | 0.87 | 0.81 | 1 | 0.95 | 0.77 |
| SocAnx_04A | 2.263 | -2.474 | -1.397 | -0.845 | 0.582 | 1.162 | 1.948 | -0.171 | 1.03 | | 1.17 | 0.94 | 0.91 | 0.99 | 0.87 | 0.61 |
| SocAnx_04B | 2.104 | -2.195 | -1.161 | -0.601 | 0.898 | 1.466 | 2.204 | 0.102 | 0.6 | | 0.87 | 0.96 | 0.94 | 0.95 | 0.82 | 0.53 |
| SocAnx_05 | 3.349 | -2.480 | -1.525 | -1.095 | 0.291 | 0.991 | 1.775 | -0.341 | 0.48 | | 0.77 | 0.92 | 0.93 | 0.94 | 0.86 | 0.59 |
| Mean | 3.014 | -2.459 | -1.465 | -1.038 | 0.464 | 1.105 | 1.958 | -0.239 |  |  |  |  |  |  |  |  |
| Test information |  |  |  |  |  |  |  |  | 5.96 | | 6.93 | 5.37 | 5.27 | 6.17 | 5.78 | 4.43 |
| SEM |  |  |  |  |  |  |  |  | 0.41 | | 0.38 | 0.43 | 0.44 | 0.4 | 0.42 | 0.48 |
| Reliability |  |  |  |  |  |  |  |  | 0.83 | | 0.86 | 0.81 | 0.81 | 0.84 | 0.83 | 0.77 |

*Model adjusted for residual correlations between 1) items 1 and 2; 2) items 4A and 4B

22

**Table S12.** Item Response Theory Parameters for E-SWAN DMDD Scale.

|  |  | Discrimination (α) and difficulty (β) parameters | | | | | |  |  | Item information by area of the latent trait (z-score) | | | | | | |
| --- | --- | --- | --- | --- | --- | --- | --- | --- | --- | --- | --- | --- | --- | --- | --- | --- |
|  | α | β1 | β2 | β3 | β4 | β5 | β6 | Mean(β) | -3 | | -2 | -1 | 0 | 1 | 2 | 3 |
| DMDD_1A | 2.234 | -1.868 | -0.927 | -0.512 | 0.660 | 1.360 | 2.189 | 0.150 | 0.69 | | 1.43 | 1.55 | 0.97 | 0.94 | 1.26 | 1.25 |
| DMDD_1B | 2.561 | -1.166 | -0.357 | 0.049 | 1.480 | 2.197 | 3.103 | 0.884 | 0.77 | | 1.47 | 1.52 | 0.97 | 1.04 | 1.34 | 1.28 |
| DMDD_1C | 2.547 | -0.969 | -0.233 | 0.104 | 1.394 | 1.949 | 2.721 | 0.828 | 0.81 | | 1.52 | 1.47 | 0.82 | 0.85 | 1.19 | 1.29 |
| DMDD_2A | 2.041 | -1.420 | -0.629 | -0.187 | 1.145 | 1.764 | 2.582 | 0.543 | 0.61 | | 1.31 | 1.56 | 1.1 | 1.08 | 1.36 | 1.24 |
| DMDD_2B | 2.641 | -0.836 | -0.111 | 0.283 | 1.755 | 2.325 | 3.009 | 1.071 | 0.97 | | 1.39 | 1.18 | 0.89 | 1.07 | 1.16 | 0.97 |
| DMDD_2C | 2.608 | -0.766 | -0.035 | 0.349 | 1.685 | 2.266 | 2.770 | 1.045 | 1 | | 1.42 | 1.14 | 0.85 | 0.98 | 1.04 | 0.93 |
| DMDD_3A | 1.987 | -1.032 | -0.216 | 0.070 | 1.235 | 1.916 | 2.648 | 0.770 | 0.99 | | 1.38 | 1.1 | 0.79 | 0.94 | 1.01 | 0.9 |
| DMDD_3B | 2.407 | -0.710 | 0.063 | 0.353 | 1.863 | 2.332 | 2.858 | 1.127 | 0.72 | | 1.31 | 1.28 | 0.9 | 0.95 | 1.12 | 1.03 |
| DMDD_3C | 2.416 | -0.715 | 0.093 | 0.384 | 1.799 | 2.211 | 2.671 | 1.074 | 0.48 | | 1.04 | 1.32 | 1.05 | 0.94 | 1.16 | 1.12 |
| DMDD_4A | 2.213 | -1.413 | -0.659 | -0.252 | 1.139 | 1.818 | 2.612 | 0.541 | 0.57 | | 1.14 | 1.35 | 1.11 | 1.14 | 1.2 | 0.93 |
| DMDD_4B | 3.144 | -0.761 | -0.136 | 0.235 | 1.582 | 2.220 | 2.869 | 1.002 | 0.71 | | 1.22 | 1.27 | 1 | 1.04 | 1.08 | 0.9 |
| DMDD_4C | 2.977 | -0.803 | -0.104 | 0.211 | 1.346 | 1.887 | 2.473 | 0.835 | 0.4 | | 0.91 | 1.25 | 1.13 | 1.04 | 1.2 | 1.08 |
| DMDD_5A | 1.931 | -1.534 | -0.819 | -0.301 | 1.040 | 1.754 | 2.499 | 0.440 | 0.79 | | 1.22 | 1.16 | 0.97 | 1.02 | 0.99 | 0.76 |
| DMDD_5B | 2.666 | -1.004 | -0.476 | -0.089 | 1.261 | 1.907 | 2.567 | 0.694 | 0.62 | | 1.11 | 1.22 | 1.06 | 1.07 | 1.05 | 0.77 |
| DMDD_5C | 2.456 | -1.106 | -0.461 | -0.104 | 1.237 | 1.847 | 2.641 | 0.676 | 0.32 | | 0.74 | 1.11 | 1.1 | 1.01 | 1.11 | 0.99 |
| DMDD_6A | 2.365 | -1.582 | -0.976 | -0.541 | 0.644 | 1.240 | 2.127 | 0.152 | 0.31 | | 0.7 | 1.07 | 1.12 | 1.06 | 1.16 | 0.99 |
| DMDD_6B | 3.117 | -1.037 | -0.471 | -0.052 | 1.301 | 2.081 | 2.995 | 0.803 | 0.59 | | 0.97 | 1.05 | 0.92 | 0.96 | 0.95 | 0.7 |
| DMDD_6C | 3.106 | -1.031 | -0.570 | -0.128 | 1.008 | 1.641 | 2.355 | 0.546 | 0.51 | | 0.85 | 1 | 0.94 | 0.95 | 0.96 | 0.75 |
| DMDD_7A | 2.250 | -1.972 | -0.772 | -0.254 | 1.556 | 2.263 | 3.127 | 0.658 | 0.57 | | 0.94 | 1.05 | 0.97 | 0.98 | 0.9 | 0.62 |
| DMDD_7B | 3.129 | -1.105 | -0.166 | 0.253 | 1.506 | 2.264 | 3.297 | 1.008 | 0.46 | | 0.81 | 0.96 | 0.84 | 0.81 | 0.92 | 0.85 |
| DMDD_7C | 2.862 | -1.076 | -0.537 | -0.190 | 0.993 | 1.699 | 2.493 | 0.564 | 0.6 | | 0.99 | 1.14 | 1.09 | 0.99 | 0.76 | 0.43 |
| DMDD_8A | 2.232 | -1.458 | -0.605 | -0.239 | 1.280 | 1.920 | 2.713 | 0.602 | 0.6 | | 0.96 | 1.1 | 1.06 | 0.96 | 0.72 | 0.41 |
| DMDD_8B | 3.548 | -0.748 | -0.211 | 0.104 | 1.309 | 1.998 | 2.504 | 0.826 | 0.57 | | 0.91 | 1.04 | 1 | 0.92 | 0.73 | 0.45 |
| DMDD_8C | 3.000 | -0.855 | -0.199 | 0.158 | 1.480 | 2.137 | 2.691 | 0.902 | 0.68 | | 0.95 | 0.98 | 0.92 | 0.84 | 0.66 | 0.4 |
| DMDD_9A | 1.854 | -1.389 | -0.574 | -0.056 | 1.498 | 2.208 | 2.952 | 0.773 | 0.51 | | 0.85 | 1.02 | 1.01 | 0.95 | 0.77 | 0.47 |
| DMDD_9B | 2.336 | -1.052 | -0.301 | 0.134 | 2.226 | 2.895 | 3.506 | 1.235 | 0.64 | | 0.93 | 1.03 | 0.98 | 0.86 | 0.63 | 0.36 |
| DMDD_9C | 2.246 | -1.183 | -0.327 | 0.160 | 1.690 | 2.321 | 3.056 | 0.953 | 0.68 | | 0.95 | 1.01 | 0.95 | 0.84 | 0.62 | 0.35 |
| DMDD_10A | 2.293 | -1.344 | -0.661 | -0.283 | 0.937 | 1.622 | 2.380 | 0.442 | 0.37 | | 0.69 | 0.96 | 1.03 | 0.97 | 0.8 | 0.51 |
| DMDD_10B | 3.269 | -0.798 | -0.354 | 0.002 | 1.238 | 1.933 | 2.495 | 0.753 | 0.4 | | 0.63 | 0.78 | 0.82 | 0.79 | 0.68 | 0.47 |
| DMDD_10C | 2.670 | -0.947 | -0.363 | 0.048 | 1.158 | 1.870 | 2.495 | 0.710 | 0.48 | | 0.7 | 0.83 | 0.84 | 0.77 | 0.6 | 0.37 |
| Mean | 2.570 | -1.123 | -0.403 | -0.010 | 1.348 | 1.995 | 2.713 | 0.753 |  |  |  |  |  |  |  |  |
| Test information |  |  |  |  |  |  |  |  | 18.41 | | 31.48 | 34.49 | 29.21 | 28.74 | 29.14 | 23.55 |
| SEM |  |  |  |  |  |  |  |  | 0.23 | | 0.18 | 0.17 | 0.19 | 0.19 | 0.19 | 0.21 |
| Reliability |  |  |  |  |  |  |  |  | 0.95 | | 0.97 | 0.97 | 0.97 | 0.97 | 0.97 | 0.96 |

23

*Model adjusted for residual correlations between A, B, and C for each item

**Table S13.** Item Response Theory Parameters for Affective Reactivity Index (ARI).

|  | Discrimination (α) and difficulty (β) parameters | | | |  | Item information by area of the latent trait (z-score) | | | | | | |
| --- | --- | --- | --- | --- | --- | --- | --- | --- | --- | --- | --- | --- |
|  | α | β1 | β2 | Mean(β) | -3 | | -2 | -1 | 0 | 1 | 2 | 3 |
| ARI_P_01 | 0.422 | -1.955 | 3.584 | 0.815 | 0.05 | | 0.17 | 0.42 | 0.58 | 0.58 | 0.49 | 0.24 |
| ARI_P_02 | 2.089 | -0.507 | 1.681 | 0.587 | 0.01 | | 0.08 | 0.8 | 1.49 | 0.3 | 0.82 | 1.48 |
| ARI_P_03 | 2.416 | 0.765 | 1.684 | 1.225 | 0.01 | | 0.03 | 0.14 | 0.43 | 0.74 | 0.77 | 0.55 |
| ARI_P_04 | 4.090 | 1.304 | 2.139 | 1.722 | 0 | | 0.01 | 0.05 | 0.17 | 0.42 | 0.65 | 0.65 |
| ARI_P_05 | 3.700 | 0.400 | 1.535 | 0.968 | 0 | | 0 | 0.01 | 0.31 | 2.4 | 0.5 | 0.15 |
| ARI_P_06 | 2.251 | -0.017 | 1.642 | 0.813 | 0 | | 0.04 | 0.33 | 1.33 | 0.68 | 0.8 | 1.22 |
| ARI_P_07 | 2.254 | 0.138 | 1.319 | 0.729 | 0.01 | | 0.04 | 0.24 | 0.89 | 0.91 | 1.01 | 0.67 |
| Mean | 2.460 | 0.018 | 1.941 | 0.979 |  |  |  |  |  |  |  |  |
| Test Info |  |  |  |  | 0.08 | | 0.37 | 1.99 | 5.19 | 6.03 | 5.05 | 4.97 |
| SEM |  |  |  |  | 3.55 | | 1.64 | 0.71 | 0.44 | 0.41 | 0.45 | 0.45 |
| Reliability |  |  |  |  | -11.62 | | -1.69 | 0.5 | 0.81 | 0.83 | 0.8 | 0.8 |

*Not adjusted for residual correlations

24

**Table S14.** Item Response Theory Parameters for Mood and Feelings Questionnaire (MFQ).

|  | Discrimination (α) and difficulty (β) parameters | | | |  | Item information by area of the latent trait (z-score) | | | | | | |
| --- | --- | --- | --- | --- | --- | --- | --- | --- | --- | --- | --- | --- |
|  | α | β1 | β2 | Mean(β) | -3 | | -2 | -1 | 0 | 1 | 2 | 3 |
| MFQ_P_01 | 2.085 | 0.137 | 1.868 | 1.003 | 0.03 | | 0.11 | 0.3 | 0.5 | 0.54 | 0.52 | 0.33 |
| MFQ_P_02 | 1.902 | 1.271 | 2.896 | 2.084 | 0.01 | | 0.03 | 0.1 | 0.24 | 0.43 | 0.52 | 0.47 |
| MFQ_P_03 | 1.148 | 1.579 | 3.487 | 2.533 | 0.04 | | 0.07 | 0.12 | 0.17 | 0.2 | 0.2 | 0.17 |
| MFQ_P_04 | 0.776 | 1.507 | 3.904 | 2.706 | 0.05 | | 0.07 | 0.09 | 0.11 | 0.12 | 0.11 | 0.1 |
| MFQ_P_05 | 1.257 | 1.369 | 2.733 | 2.051 | 0.04 | | 0.08 | 0.14 | 0.23 | 0.29 | 0.28 | 0.21 |
| MFQ_P_06 | 1.489 | 1.731 | 3.290 | 2.511 | 0.02 | | 0.05 | 0.11 | 0.19 | 0.28 | 0.32 | 0.28 |
| MFQ_P_07 | 1.342 | 0.820 | 2.493 | 1.657 | 0.05 | | 0.09 | 0.16 | 0.24 | 0.28 | 0.26 | 0.19 |
| MFQ_P_08 | 2.738 | 1.206 | 2.557 | 1.882 | 0 | | 0.01 | 0.06 | 0.2 | 0.49 | 0.63 | 0.63 |
| MFQ_P_09 | 1.710 | 1.379 | 2.876 | 2.128 | 0.02 | | 0.05 | 0.11 | 0.24 | 0.39 | 0.45 | 0.37 |
| MFQ_P_10 | 1.302 | 0.431 | 2.308 | 1.370 | 0.05 | | 0.11 | 0.19 | 0.28 | 0.31 | 0.27 | 0.19 |
| MFQ_P_11 | 1.442 | -0.401 | 1.747 | 0.673 | 0.08 | | 0.16 | 0.27 | 0.34 | 0.34 | 0.26 | 0.16 |
| MFQ_P_12 | 1.503 | 1.130 | 2.914 | 2.022 | 0.02 | | 0.06 | 0.14 | 0.27 | 0.4 | 0.43 | 0.34 |
| MFQ_P_13 | 1.510 | 2.574 | 3.859 | 3.217 | 0.02 | | 0.03 | 0.07 | 0.14 | 0.23 | 0.31 | 0.32 |
| MFQ_P_14 | 1.355 | 1.273 | 2.801 | 2.037 | 0.03 | | 0.07 | 0.14 | 0.24 | 0.32 | 0.32 | 0.24 |
| MFQ_P_15 | 2.515 | 1.499 | 2.487 | 1.993 | 0 | | 0.01 | 0.05 | 0.16 | 0.42 | 0.68 | 0.67 |
| MFQ_P_16 | 2.615 | 1.810 | 2.906 | 2.358 | 0 | | 0.01 | 0.03 | 0.11 | 0.29 | 0.53 | 0.62 |
| MFQ_P_17 | 1.903 | 1.928 | 3.131 | 2.530 | 0.01 | | 0.03 | 0.07 | 0.16 | 0.3 | 0.41 | 0.4 |
| MFQ_P_18 | 2.519 | 1.897 | 3.486 | 2.692 | 0.01 | | 0.01 | 0.04 | 0.11 | 0.25 | 0.39 | 0.44 |
| MFQ_P_19 | 2.388 | 2.123 | 3.638 | 2.881 | 0.01 | | 0.02 | 0.04 | 0.1 | 0.22 | 0.36 | 0.43 |
| MFQ_P_20 | 1.770 | 2.039 | 3.478 | 2.759 | 0.01 | | 0.03 | 0.08 | 0.15 | 0.27 | 0.35 | 0.35 |
| MFQ_P_21 | 1.188 | 0.460 | 2.377 | 1.419 | 0.06 | | 0.11 | 0.17 | 0.23 | 0.25 | 0.22 | 0.16 |
| MFQ_P_22 | 1.786 | 1.539 | 3.055 | 2.297 | 0.01 | | 0.04 | 0.09 | 0.21 | 0.36 | 0.44 | 0.4 |
| MFQ_P_23 | 3.357 | 1.462 | 2.537 | 2.000 | 0 | | 0 | 0.02 | 0.08 | 0.32 | 0.72 | 0.74 |
| MFQ_P_24 | 2.294 | 1.398 | 2.849 | 2.124 | 0.01 | | 0.03 | 0.08 | 0.2 | 0.38 | 0.5 | 0.49 |
| MFQ_P_25 | 1.799 | 1.606 | 2.921 | 2.264 | 0.01 | | 0.03 | 0.09 | 0.2 | 0.38 | 0.49 | 0.44 |
| MFQ_P_26 | 1.101 | 1.817 | 3.531 | 2.674 | 0.04 | | 0.07 | 0.1 | 0.14 | 0.16 | 0.17 | 0.14 |
| MFQ_P_27 | 1.472 | 1.303 | 3.132 | 2.218 | 0.03 | | 0.06 | 0.13 | 0.23 | 0.33 | 0.36 | 0.29 |
| MFQ_P_28 | 2.098 | 1.454 | 2.897 | 2.176 | 0.01 | | 0.03 | 0.08 | 0.2 | 0.39 | 0.52 | 0.5 |
| MFQ_P_29 | 1.554 | 0.716 | 2.363 | 1.540 | 0.03 | | 0.08 | 0.18 | 0.32 | 0.41 | 0.39 | 0.27 |
| MFQ_P_30 | 1.755 | 0.969 | 2.499 | 1.734 | 0.02 | | 0.06 | 0.15 | 0.3 | 0.44 | 0.46 | 0.34 |
| MFQ_P_31 | 2.205 | 1.112 | 3.118 | 2.115 | 0.01 | | 0.03 | 0.1 | 0.24 | 0.41 | 0.44 | 0.44 |
| MFQ_P_32 | 1.118 | 1.377 | 3.886 | 2.632 | 0.04 | | 0.07 | 0.12 | 0.16 | 0.2 | 0.2 | 0.17 |
| MFQ_P_33 | 1.066 | 1.834 | 3.713 | 2.774 | 0.04 | | 0.07 | 0.1 | 0.14 | 0.17 | 0.17 | 0.15 |
| MFQ_P_34 | 2.245 | 1.384 | 2.548 | 1.966 | 0 | | 0.02 | 0.06 | 0.2 | 0.47 | 0.66 | 0.62 |

25

| Mean | 1.774 | 1.345 | 2.950 | 2.147 |  |  |  |  |  |  |
| --- | --- | --- | --- | --- | --- | --- | --- | --- | --- | --- |
| Test Info |  |  |  | 0.85 | 1.82 | 3.78 | 7.05 | 11.05 | 13.35 | 12.04 |
| SEM |  |  |  | 1.09 | 0.74 | 0.51 | 0.38 | 0.3 | 0.27 | 0.29 |
| Reliability |  |  |  | -0.18 | 0.45 | 0.74 | 0.86 | 0.91 | 0.93 | 0.92 |

*Not adjusted for residual correlations

**Table S15.** Item Response Theory Parameters for Screen for Child Anxiety and Related Disorders (SCARED) Panic Disorder.

|  | Discrimination (α) and difficulty (β) parameters | | | |  | Item information by area of the latent trait (z-score) | | | | | | |
| --- | --- | --- | --- | --- | --- | --- | --- | --- | --- | --- | --- | --- |
|  | α | β1 | β2 | Mean(β) | -3 | | -2 | -1 | 0 | 1 | 2 | 3 |
| SCARED_P_01 | 1.091 | 2.269 | 2.133 | 2.201 | 0 | | 0.01 | 0.05 | 0.17 | 0.48 | 0.76 | 0.73 |
| SCARED_P_06 | 2.122 | 2.646 | 3.500 | 3.073 | 0 | | 0 | 0 | 0 | 0 | 0.02 | 0.17 |
| SCARED_P_09 | 2.374 | 5.053 | 1.072 | 3.063 | 0.02 | | 0.04 | 0.07 | 0.14 | 0.23 | 0.3 | 0.31 |
| SCARED_P_12 | 1.770 | 2.901 | 1.473 | 2.187 | 0.01 | | 0.02 | 0.06 | 0.16 | 0.38 | 0.6 | 0.56 |
| SCARED_P_15 | 3.051 | 4.998 | 0.960 | 2.979 | 0.02 | | 0.04 | 0.08 | 0.14 | 0.21 | 0.27 | 0.26 |
| SCARED_P_18 | -0.002 | 1.739 | 1.808 | 1.774 | 0.05 | | 0.11 | 0.21 | 0.32 | 0.36 | 0.31 | 0.21 |
| SCARED_P_19 | 1.244 | 2.688 | 1.971 | 2.330 | 0.01 | | 0.02 | 0.08 | 0.21 | 0.44 | 0.6 | 0.54 |
| SCARED_P_22 | 1.888 | 3.092 | 1.416 | 2.254 | 0.02 | | 0.04 | 0.09 | 0.18 | 0.3 | 0.38 | 0.34 |
| SCARED_P_24 | 1.932 | 3.727 | 0.993 | 2.360 | 0.03 | | 0.06 | 0.11 | 0.19 | 0.27 | 0.3 | 0.25 |
| SCARED_P_27 | 2.084 | 2.782 | 2.913 | 2.848 | 0 | | 0 | 0 | 0.01 | 0.07 | 0.3 | 0.83 |
| SCARED_P_30 | 1.484 | 2.608 | 1.887 | 2.248 | 0 | | 0 | 0.02 | 0.07 | 0.3 | 0.78 | 0.97 |
| SCARED_P_34 | 1.806 | 2.847 | 1.529 | 2.188 | 0.01 | | 0.02 | 0.05 | 0.15 | 0.35 | 0.56 | 0.56 |
| SCARED_P_38 | 2.063 | 3.144 | 1.765 | 2.455 | 0 | | 0 | 0.01 | 0.04 | 0.15 | 0.52 | 0.94 |
| Mean | 1.762 | 3.115 | 1.802 | 2.458 |  |  |  |  |  |  |  |  |
| Test Info |  |  |  |  | 0.15 | | 0.36 | 0.82 | 1.79 | 3.56 | 5.7 | 6.68 |
| SEM |  |  |  |  | 2.54 | | 1.66 | 1.1 | 0.75 | 0.53 | 0.42 | 0.39 |
| Reliability |  |  |  |  | -5.45 | | -1.76 | -0.21 | 0.44 | 0.72 | 0.82 | 0.85 |

*Not adjusted for residual correlations

26

**Table S16.** Item Response Theory Parameters for Screen for Child Anxiety and Related Disorders (SCARED) Social Anxiety.

|  | Discrimination (α) and difficulty (β) parameters | | | |  | Item information by area of the latent trait (z-score) | | | | | | |
| --- | --- | --- | --- | --- | --- | --- | --- | --- | --- | --- | --- | --- |
|  | α | β1 | β2 | Mean(β) | -3 | | -2 | -1 | 0 | 1 | 2 | 3 |
| SCARED_P_03 | -0.198 | 1.399 | 1.874 | 1.637 | 0.06 | | 0.15 | 0.32 | 0.47 | 0.49 | 0.38 | 0.2 |
| SCARED_P_10 | 0.268 | 1.807 | 2.165 | 1.986 | 0.02 | | 0.07 | 0.22 | 0.47 | 0.58 | 0.57 | 0.39 |
| SCARED_P_26 | 0.248 | 1.274 | 3.533 | 2.404 | 0 | | 0.02 | 0.13 | 0.82 | 1.11 | 0.83 | 1.19 |
| SCARED_P_32 | -0.189 | 0.998 | 4.152 | 2.575 | 0.01 | | 0.11 | 0.94 | 1.24 | 0.31 | 1.07 | 1.11 |
| SCARED_P_39 | 0.365 | 1.454 | 1.596 | 1.525 | 0.05 | | 0.11 | 0.23 | 0.36 | 0.41 | 0.33 | 0.19 |
| SCARED_P_40 | 0.284 | 1.598 | 1.847 | 1.723 | 0.04 | | 0.1 | 0.23 | 0.39 | 0.47 | 0.39 | 0.23 |
| SCARED_P_41 | -0.026 | 1.466 | 2.517 | 1.992 | 0.03 | | 0.1 | 0.34 | 0.62 | 0.63 | 0.64 | 0.39 |
| Mean | 0.107 | 1.428 | 2.526 | 1.977 |  |  |  |  |  |  |  |  |
| Test Info |  |  |  |  | 0.21 | | 0.67 | 2.41 | 4.38 | 4.01 | 4.21 | 3.71 |
| SEM |  |  |  |  | 2.21 | | 1.22 | 0.64 | 0.48 | 0.5 | 0.49 | 0.52 |
| Reliability |  |  |  |  | -3.87 | | -0.49 | 0.59 | 0.77 | 0.75 | 0.76 | 0.73 |

*Model adjusted for residual correlations between 1) items 39 and 40; 2) items 03 and 10

**Table S17.** Measurement Invariance between HBN and PA sample on Social Anxiety and Depression E-SWAN Scales*.*

These values indicate that the same latent trait is being measured in both samples.

|  | **CFI** | **RMSEA** |
| --- | --- | --- |
| **E-SWAN Social Anxiety** | 0.001 | 0.012 |
| **E-SWAN Depression** | 0.001 | 0.003 |

27
